# Reactant enrichment in hollow void of Pt NPs@MnOx nanoreactors for boosting hydrogenation performance

**DOI:** 10.1093/nsr/nwad201

**Published:** 2023-07-20

**Authors:** Yanfu Ma, Liwei Wang, Wantong Zhao, Tianyi Liu, Haitao Li, Wenhao Luo, Qike Jiang, Wei Liu, Qihua Yang, Jun Huang, Riguang Zhang, Jian Liu, G Q Max Lu, Can Li

**Affiliations:** State Key Laboratory of Catalysis, Dalian Institute of Chemical Physics (DICP), Chinese Academy of Sciences, Dalian116023, China; State Key Laboratory of Catalysis, Dalian Institute of Chemical Physics (DICP), Chinese Academy of Sciences, Dalian116023, China; Center of Materials Science and Optoelectronics Engineering, University of Chinese Academy of Sciences, Beijing100049, China; State Key Laboratory of Clean and Efficient Coal Utilization, Taiyuan University of Technology, Taiyuan030024, China; State Key Laboratory of Catalysis, Dalian Institute of Chemical Physics (DICP), Chinese Academy of Sciences, Dalian116023, China; DICP-Surrey Joint Centre for Future Materials, Department of Chemical and Process Engineering, University of Surrey, GuildfordGU2 7XH, UK; State Key Laboratory of Catalysis, Dalian Institute of Chemical Physics (DICP), Chinese Academy of Sciences, Dalian116023, China; CAS Key Laboratory of Science and Technology on Applied Catalysis, Dalian Institute of Chemical Physics, Chinese Academy of Sciences, Dalian116023, China; School of Chemistry and Chemical Engineering, Inner Mongolia University, Hohhot010021, China; Division of Energy Research Resources, Dalian National Laboratory for Clean Energy, Dalian Institute of Chemical Physics, Chinese Academy of Sciences, Dalian116023, China; Division of Energy Research Resources, Dalian National Laboratory for Clean Energy, Dalian Institute of Chemical Physics, Chinese Academy of Sciences, Dalian116023, China; Key Laboratory of the Ministry of Education for Advanced Catalysis Materials, Zhejiang Key Laboratory for Reactive Chemistry on Solid Surfaces, Institute of Physical Chemistry, Zhejiang Normal University, Jinhua 321004, China; Laboratory for Catalysis Engineering, School of Chemical and Biomolecular Engineering, Sydney Nano Institute, The University of Sydney, Sydney2006, Australia; State Key Laboratory of Clean and Efficient Coal Utilization, Taiyuan University of Technology, Taiyuan030024, China; State Key Laboratory of Catalysis, Dalian Institute of Chemical Physics (DICP), Chinese Academy of Sciences, Dalian116023, China; Center of Materials Science and Optoelectronics Engineering, University of Chinese Academy of Sciences, Beijing100049, China; DICP-Surrey Joint Centre for Future Materials, Department of Chemical and Process Engineering, University of Surrey, GuildfordGU2 7XH, UK; School of Chemistry and Chemical Engineering, Inner Mongolia University, Hohhot010021, China; University of Surrey, GuildfordGU2 7XH, UK; State Key Laboratory of Catalysis, Dalian Institute of Chemical Physics (DICP), Chinese Academy of Sciences, Dalian116023, China

**Keywords:** reactant enrichment, nanoreactor effect, selective hydrogenation, hollow nanostructure, mesoporous materials

## Abstract

In confined mesoscopic spaces, the unraveling of a catalytic mechanism with complex mass transfer and adsorption processes such as reactant enrichment is a great challenge. In this study, a hollow nanoarchitecture of MnO_x_-encapsulated Pt nanoparticles was designed as a nanoreactor to investigate the reactant enrichment in a mesoscopic hollow void. By employing advanced characterization techniques, we found that the reactant-enrichment behavior is derived from directional diffusion of the reactant driven through the local concentration gradient and this increased the amount of reactant. Combining experimental results with density functional theory calculations, the superior cinnamyl alcohol (COL) selectivity originates from the selective adsorption of cinnamaldehyde (CAL) and the rapid formation and desorption of COL in the MnO_x_ shell. The superb performance of 95% CAL conversion and 95% COL selectivity is obtained at only 0.5 MPa H_2_ and 40 min. Our findings showcase that a rationally designed nanoreactor could boost catalytic performance in chemoselective hydrogenation, which can be of great aid and potential in various application scenarios.

## Teaser

Hollow nanoarchitecture MnOx encapsulated Pt nanoparticles was designed as nanoreactor to investigate the reactant enrichment in a mesoscopic hollow void and their contributions to selective hydrogenation of cinnamaldehyde toward cinnamyl alcohol.

## INTRODUCTION

The rapid development of modern processing and manufacturing industries has been propelled by breakthroughs and innovations in catalysis, which affords >80% of industrial production of fuels, fine chemicals and pharmaceuticals by hydrogenation, oxidation and free radical reactions [1–3]. Hollow-structured supported metal catalysts (i.e. nanoreactor catalysts) with encapsulated active sites and well-defined shells provide an ideal place for multicomponents to react or transform cooperatively in an orderly manner and efficiently have been recognized as one of the most popular catalyst candidates. The synthesis of hollow catalytic nanoreactors is mainly performed through a top-down or bottom-up strategy. The hollow structure encapsulated with metal nanoparticles (NPs) is fabricated by using organic or inorganic matrices as templates in a liquid medium and then removing them by selective etching or calcination [4–6]. While it is true that those strategies have reached a level of maturity that allows the creation of aesthetically interesting structures (nanoflowers, nanoboxes, multishelled nanospheres, etc.), the catalytic theoretical development of nanoreactors is still not comprehensive enough.

Although reactant enrichment has been proposed based on nanostructured catalysts by investigating the relationship between the catalytic performance and the structure of nanoreactors, it remains scarce at the mesoscale level (500–2000 nm) [5,7–11]. At the nanoscale level, nanoreactors internally loaded with metals exhibit enhanced catalytic activity relative to externally loaded ones. For example, superior catalytic activity could be achieved for styrene hydrogenation where PdCu particles were encapsulated inside the hollow nanocarbon shell as opposed to being dispersed on the external surface of the shell [11]. Similarly, this phenomenon has also been observed over other nanostructured catalysts by tuning the spatial location of active metals, such as Pt@CNTs, Ru@HCSs, etc. [12,13]. Concurrently, a simple model for hydrogen adsorption by hollow silica was established for the in-depth understanding of reactant enrichment [14]. Density functional theory (DFT) calculation results showed that as the surface curvature of hollow SiO_2_ increases, the adsorption capacity for hydrogen gradually rises, which enables hydrogen to be more easily enriched inside the void, in good agreement with the hydrogen adsorption experiment. However, two issues require consideration. One is that different synthetic methods or sequences are needed to achieve the loading of active metals inside and outside the hollow nanostructure, which impacts the microenvironment around the active sites, as well as the essential active sites. Furthermore, this approach fails to explain and delve into the origin of the enrichment effect at the mesoscale level, and neither did adjusting the size of the void. Another problem is that the reactant enrichment at the mesoscale level involves many processes such as adsorption and diffusion, which cannot be elaborated by constructing simple computational models at the nanoscale level. Therefore, investigation of the reactant enrichment at the mesoscale level requires maintaining the intrinsic active sites constant when constructing the research model both with or without enrichment behavior.

Controlling selectivity is a pertinent task in catalysis, especially when several unsaturated functional groups coexist. In the selective hydrogenation of cinnamaldehyde (CAL), hydrogenation of the C=C group is thermodynamically favored to the C=O group. The achievement of high selectivity needs nanoengineering to introduce organic molecules or metal oxide to improve the adsorption for the C=O group, which requires complicated synthetic steps and a balance between multiple components [15–18]. Herein, we fabricate a highly efficient nanoreactor catalyst (Pt NPs@MnO_x_) with uniformly dispersed Pt nanoparticles encapsulated in an oxygen vacancy-rich MnO_x_ hollow structure to catalyse the selective hydrogenation of CAL and investigate reactant enrichment at the mesoscale level. The catalytic performance for CAL-selective hydrogenation on Pt NPs@MnO_x_ is 3.4-fold higher than that of Pt NPs&MnO_x_ where hollow MnO_x_ has been crushed. The presence of a hollow structure was demonstrated to be critical for reactant enrichment. A series of experiments, characterizations and DFT calculations revealed that reactant enrichment is derived from the directional diffusion of reactant driven through a local concentration gradient and an increased amount of reactant adsorbed due to the enhanced adsorption ability in hollow MnO_x_. This enables Pt NPs@MnO_x_ catalyst to exhibit extremely high catalytic activities and selectivity in a wide range of reaction pressures.

## RESULTS AND DISCUSSION

### Structural characterization of Pt NPs@MnO_x_ nanoreactors

**Figure 1.**
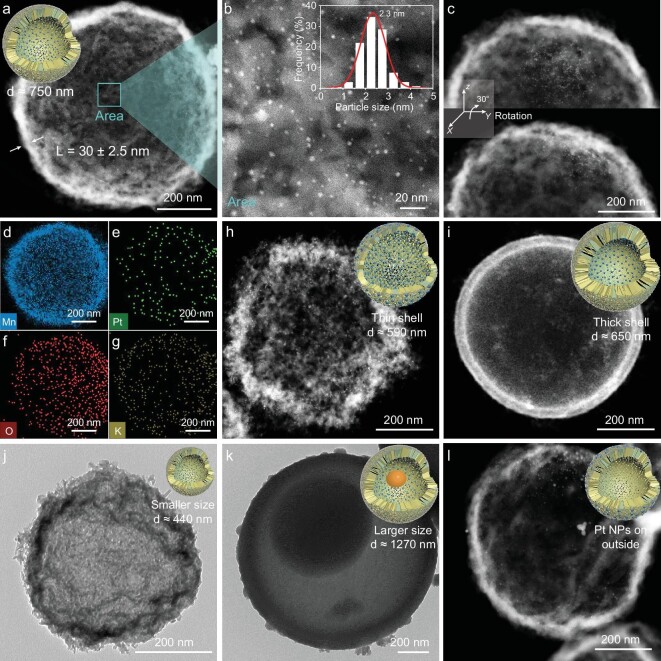
Morphology of various mesoscopic nanoreactors. (a and b) Aberration-corrected HAADF–STEM images of Pt NPs@MnO_x_ at (a) low and (b) high magnifications. The inset in (b) shows the corresponding size histograms of Pt NPs. (c) Rotation of Pt NPs@MnO_x_ was observed during HAADF–STEM measurements. (d–g) Corresponding energy-dispersive X-ray spectroscopy elemental mapping images of Mn, Pt, O and K. (h–l) HAADF–STEM or TEM images of Pt NPs@MnO_x_-Tn, Pt NPs@MnO_x_-Tk, Pt NPs@MnO_x_-S, Pt NPs@MnO_x_-L and Pt NPs/MnO_x_, respectively. In (k), APF was not completely decomposed under the same conditions because APF-Large is too big.

Initially, a hollow-structured Pt NPs@MnO_x_ nanoreactor with a uniform size of ∼750 nm in diameter and a shell thickness of ∼30 nm (Fig. 1a) was constructed through a sacrificial template strategy via the utilization of aminophenol–formaldehyde (APF) resins as the hard template (Supplementary Figs S1–S6). As illustrated in Fig. 1**b**, numerous Pt NPs with a narrow size distribution (∼2.3 nm by surveying 200 particles) can be clearly observed in a high-magnification area from an aberration-corrected high-angle annular dark-field scanning transmission electron microscopy (HAADF–STEM) image. To confirm the location of Pt NPs in the shell, HAADF–STEM images of Pt NPs@MnO_x_ catalyst are recorded before and after rotating the sample holder by 30^o^ of rotation. As shown in Fig. 1c, Pt NPs are rotated with the shell, maintaining the relative position between the Pt particles and the MnO_x_ shell well, implying that the Pt particles are fixed inside the hollow MnO_x_ shell. Energy-dispersive X-ray spectroscopy elemental mapping reveals homogeneous elemental distributions of Pt, Mn, O and K in Fig. 1d**–**g, which provides clear evidence for the formation of the Pt NPs@MnO_x_ nanoreactor. Concurrently, it was demonstrated that some structural parameters of the mesoscopic nanoreactors such as the diameter, shell thickness and stack density of the MnO_x_ shell as well as the location of the Pt NPs can be easily tailored by multiscale process engineering including adjusting the size of the APF and various synthetic conditions of the shell (Fig. 1h**–l** and Supplementary Figs S7–S9).

### The function and characterization of reactant enrichment

**Figure 2.**
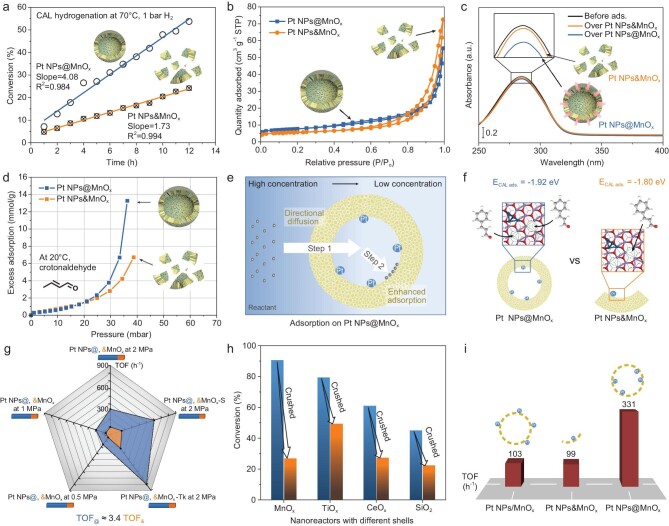
Nanoreactor effect in liquid catalytic conversion. (a) Hydrogenation performance of CAL at 70^o^C, 1 bar H_2_. (b–d) N_2_ adsorption–desorption isotherms at 77 K (b), UV–vis spectrum of CAL adsorption experiment (c), *in situ* gravimetric adsorption analysis profiles (d) over Pt NPs@MnO_x_ and Pt NPs&MnO_x_, respectively. (e) Schematic illustration of reactant enrichment on Pt NPs@MnO_x_. (f) The adsorption energy of one CAL adsorbed at one O_V_ site and that of two CAL adsorbed at two O_V_ sites on Pt NPs&MnO_x_ with two O_V_ sites. (g) Activity comparison over a series of nanoreactors and crushed samples at 70^o^C, 10 min and various H_2_ pressures. The ratio of the lengths of the blue and orange bars represents K_@_. (h) The influences of different shells. Experimental conditions: 70^o^C, 15 min and 2 MPa H_2_ for MnO_x_; 70^o^C, 3 h and 2 MPa H_2_ for TiO_x_; 70^o^C, 6 h and 2 MPa H_2_ for CeO_x_; 40^o^C, 10 min and 2 MPa H_2_ for SiO_2_ with the 3-fold reactant. (i) TOF of Pt NPs/MnO_x_, Pt NPs&MnO_x_ and Pt NPs@MnO_x_. TOF is calculated by the mole number of converted CAL (mole number of surface Pt)^−1^ h^−1^ after reaction at 70^o^C, 10 min and 2 MPa H_2_.

The catalytic activity of Pt NPs@MnO_x_ was evaluated by hydrogenation of CAL. Noticeably, CAL could be hydrogenated at 70^o^C and 1 bar H_2_ in Fig. 2a. Commonly, this activity is partly attributed to the reactant enrichment in the confined space. To prove it, Pt NPs@MnO_x_ was physically crushed into an open structure (denoted as Pt NPs&MnO_x_) (transmission electron microscopy (TEM) images are in Supplementary Fig. S10) to ensure consistency across all physicochemical characteristics between them except for the hollow structure. In Fig. 2a, Pt NPs&MnO_x_ presents low catalytic activity. The CAL conversion on Pt NPs@MnO_x_ is twice as high as that on Pt NPs&MnO_x_, even though an open structure can significantly facilitate the accessibility of the active sites, highlighting the significant structural superiority of the nanoreactor. In Fig. 2b, nitrogen adsorption–desorption isotherms for both Pt NPs@MnO_x_ and Pt NPs&MnO_x_ display a type Ⅳ shape with a similar specific surface area, indicating the existence of a mesoporous structure for mass transfer. Hence, the effect of the specific surface area on adsorption can be excluded (Supplementary Table 1). Then, a CAL adsorption experiment is performed by using UV–vis measurement in which the adsorption amount of CAL from the ethanol solution is evaluated [12]. As displayed in Fig. 2c, a 3.5% decrease in the CAL intensity is observed for Pt NPs@MnO_x_, which is nearly 6-fold that of Pt NPs&MnO_x_ with a 0.6% decrease, implying that the hollow MnO_x_ shell enables some CAL (∼0.156 μg_CAL_/mg_cat_) to be drawn in. These results are in good accordance with *in situ* Fourier transform-infrared (FTIR) observation that a hollow MnO_x_ shell of Pt NPs@MnO_x_ leads to higher CAL uptake (Supplementary Fig. S11). Owing to the low saturated vapor pressure of CAL, crotonaldehyde is selected to quantify its adsorption behavior by using *in situ* gravimetric adsorption (IGA) analysis. Note that the adsorption amount for crotonaldehyde on Pt NPs@MnO_x_ is much higher than that of Pt NPs&MnO_x_, especially for pressures of >18 mbar (Fig. 2d). Combining the results of the UV–vis, *in situ* FTIR and IGA measurements, the hollow structure of the nanoreactor can absorb more reactants with an enhanced concentration. The mechanism behind this phenomenon may consist of two steps, as illustrated in Fig. 2e. As the hollow structure creates a confined space, outer reactants would continuously diffuse into the interior of the hollow structure directionally driven by the concentration gradient and/or capillary-like effect (**Step 1**). Then, these reactants are fixed on the inner surface by adsorption to keep the local low concentration in the confined space. In contrast, Pt NPs&MnO_x_ could not support this directional diffusion process. Moreover, DFT results reveal that the oxygen vacancy (O_V_) is the main adsorption site for CAL after numerous optimizations of the different initial adsorption configurations (Supplementary Table 2). Further, two O_V_ sites on the MnO_x_ shell are selected to refine the adsorption process. Interestingly, on Pt NPs&MnO_x_ with two O_V_ sites, the adsorption energy of one CAL adsorbed at one O_V_ site is –1.80 eV, and that of two CAL adsorbed at two O_V_ sites increases to –1.92 eV on Pt NPs@MnO_x_, as illustrated in Fig. 2f, proving that CAL is more strongly adsorbed on the surface of Pt NPs@MnO_x_ under excess reactants (**Step 2**). In this sense, the continuously directional diffusion to the adsorption site and enhanced adsorption strength of CAL during the complex mass transfer and adsorption processes contribute to the reinforced adsorption behavior in the confined mesoscopic space, enabling easier activation and reaction on the active sites of the Pt NPs@MnO_x_ nanoreactor.

In addition to the infiltration and adsorption mechanisms mentioned above, it needs to be considered whether the reactants will get away from the nanoreactor quickly via the channel/pore. We simulate this situation by physically mixing the nanoreactors with iron oxide (Fe_2_O_3_) and monitoring the reduction in Fe_2_O_3_ by using H_2_-TPR–MS (temperature programmed reduction coupled with mass spectrometry) (Supplementary Fig. S12a–c). The peak corresponding to the reduction of Fe^3+^ to Fe^2+^ shifts from 361^o^C to 348^o^C after mixing Pt NPs&MnO_x_ with Fe_2_O_3_, indicating that dissociated hydrogen migrates directly to the Fe_2_O_3_ surface and accelerates its reduction. However, the peak shifts to 393^o^C over the mixture of Pt NPs@MnO_x_ and Fe_2_O_3_, implying that H_2_ is preferentially enriched in the mesoscale cavities and the ensuing dissociated hydrogen needs to pass through the MnO_x_ shell to participate in the reduction of Fe_2_O_3_. Therefore, the rate of molecules leaving the nanoreactor is controlled by the shell. Finite-element simulation results also demonstrate that the nanoreactor creates a stable space with a high concentration and low flow rate to prevent the escape of dissociated hydrogen (Supplementary Fig. S12d and e). This phenomenon is also applicable to explain the enrichment of CAL, which matches the results of the UV–vis and IGA measurements.

### The quantification of the nanoreactor effect

Subsequently, we attempted to quantify the relationship between the catalytic performance and the nanoreactor effect by reactant enrichment. The influence of hydrogen pressure on the nanoreactor effect is examined using Pt NPs@MnO_x_ and crushed Pt NPs&MnO_x_ as a typical model (Supplementary Fig. S13). Notably, hydrogen pressure does not affect their initial rate in CAL hydrogenation. Comparison of the shell thickness and diameter of nanoreactors on the CAL conversion is also considered (Supplementary Figs S7, S8, S14 and S15a). As the shell thickness and diameter vary from 15–55 nm and 380–1400 nm, respectively, the CAL conversion displays a dual volcano trend (Supplementary Fig. S15b). It has been well investigated experimentally and computationally that a balancing effect exists between adsorption and diffusion [14]. These two opposing behaviors result in a volcano trend. Therefore, a nanoreactor with a shell thickness of ∼30 nm and a diameter of ∼750 nm shows the highest conversion, possessing the optimal adsorption enhancement capacity. With a shorter calcination time (≤2 h), undecomposed APF core inside the hollow structure or unsuitable Pt–Mn interaction causes a significant decrease in conversion (Supplementary Fig. S15c). To eliminate those factors, three nanoreactors (Pt NPs@MnO_x_, Pt NPs@MnO_x_–S for a small nanoreactor and Pt NPs@MnO_x_–Tk for a thick-shell nanoreactor) and their corresponding crushed samples are chosen to quantify the nanoreactor effect. Herein, we introduce the concept of the nanoreactor effect coefficient (K_@_), which reflects the catalytic performance due to the presence of a hollow structure:


\begin{eqnarray*}{{\mathrm{K}}}_@ = \frac{{{\mathrm{TO}}{{\mathrm{F}}}_@}}{{{\mathrm{TO}}{{\mathrm{F}}}_\& }} = \frac{{{\mathrm{TO}}{{\mathrm{F}}}_\& + {\mathrm{TOF}}{{\mathrm{^{\prime}}}}_@}}{{{\mathrm{TO}}{{\mathrm{F}}}_\& }},\end{eqnarray*}

where TOF_@_ is assigned to the nanoreactor activity, TOF_&_ is designated as the intrinsic activity on the crushed sample and TOFʹ_@_ is the additional activity from the increased reactant concentration caused by the hollow structure. With the crushing of the MnO_x_ shell, the catalytic performance drops obviously at 2 MPa H_2_. The K_@_ is 3.3, 3.9 and 3.6 for Pt NPs@MnO_x_, Pt NPs@MnO_x_–S and Pt NPs@MnO_x_–Tk, respectively. As the hydrogen pressure varies from 2 to 1 and 0.5 MPa, the K_@_ is slightly changed (3.1 and 3.4, respectively), as displayed in Fig. 2g. It is found that the K_@_ value fluctuates between 3 and 4, and the average value is ∼3.4 based on the current model and activity evaluation results. A more accurate value of K_@_ could be obtained with a large sample of repeated experiments to eliminate errors in synthesis and testing. When the shell consists of TiO_x_, CeO_x_ and SiO_2_, their CAL conversions decrease to different extents after crushing treatment (Fig. 2h and Supplementary Fig. S16). To study the contribution of a hollow void in the nanoreactor, apart from crushing the sample, an outer-loaded catalyst was synthesized. Although the particle sizes of Pt NPs and microenvironments are different between Pt NPs/MnO_x_ and Pt NPs&MnO_x_, they show similar TOF values, as shown in Fig. 2i. This phenomenon indicates that the differences in the microenvironment have a negligible influence on the catalytic activities. Given the phenomenon that the Pt NPs/MnO_x_ and Pt NPs&MnO_x_ possess the same open structure around Pt NPs, it is assumed that the reactants enrichment from the hollow void of Pt NPs@MnO_x_ plays a more predominant role than other factors.

### The mechanism of the nanoreactor in selective hydrogenation

**Figure 3.**
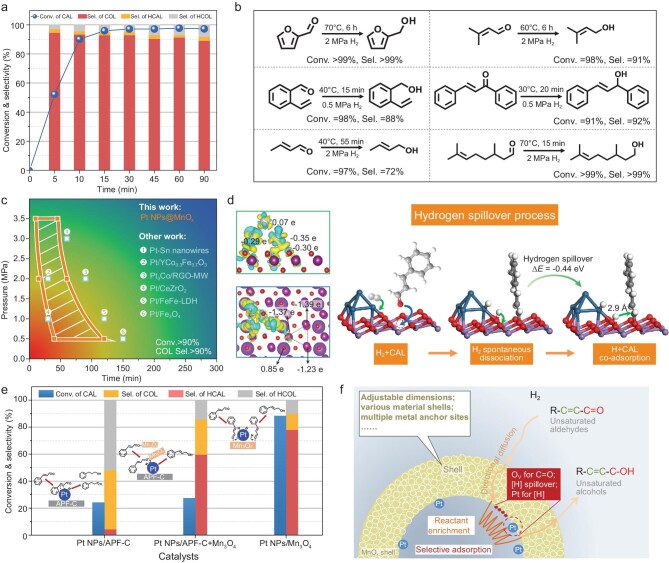
Catalytic performance and mechanism of the Pt NPs@MnO_x_ nanoreactor. (a) Curves of CAL conversion and the product distribution over Pt NPs@MnO_x_ at 70^o^C and 2 MPa H_2_. (b) Substrate scope of various unsaturated aldehydes. (c) Performance comparison over Pt-based catalyst with high conversion (>90%) and high selectivity (>90%) [19–24]. (d) H-atom spillover from Pt_4_ cluster to K_2_Mn_4_O_8_ surface on the Pt_4_/K_2_Mn_4_O_8_ catalyst. Differential charge density of surface Pt atoms on the Pt_4_/K_2_Mn_4_O_8_ from the side (side view) and top (top view) views. (e) Conversion of CAL and the product distribution over Pt NPs/APF-C, Pt NPs/Mn_3_O_4_ and their mixture at 70^o^C, 6 h and 2 MPa H_2_. (f) The reaction mechanism of the nanoreactor.

In a CAL-selective hydrogenation reaction, the formation of COL is relatively challenging because C=C hydrogenation is thermodynamically superior to C=O hydrogenation [25,26]. Unlike their differences in activity, the COL selectivity is relatively similar over Pt NPs@MnO_x_ and Pt NPs&MnO_x_ (93% versus 91% in Supplementary Fig. S17a and b), implying that the nanoreactor effect does not significantly affect the selectivity, but the activity, which is different from the confinement effect. It is well known that the confinement effect has been proposed based on nanostructured catalysts with well-defined pores and cavities at molecular dimensions, such as carbon nanotubes, zeolites, metal-organic frameworks and covalent organic frameworks [27–31]. The main function of pores is the shape- and size-selective arrangement of reactants in nanochannels, which regulates the selectivity [32–34]. However, the nanoreactor effect involves a larger range of sizes from nanoscale to mesoscale at least. The hollow void induces the reactant-enrichment behavior, which could enhance the reaction activity. Figure 3a shows that the COL selectivity is 95% over Pt NPs@MnO_x_ in the initial stage, remains at >90% after extending the reaction time to 60 min and drops to 89% after 90 min. When COL is used as the reactant, its conversion is only 30% under identical conditions (70^o^C, 2 MPa H_2_, 10 min), which is much lower than that of CAL, as shown in Supplementary Fig. S17c. This phenomenon could be well explained by DFT calculation of the adsorption energy, as shown in Supplementary Tables 2 and 3. The aldehyde group (–2.27 eV) of CAL is more easily adsorbed than the hydroxyl group of COL (–1.21 eV) on the O_V_ site of the MnO_x_ shell, indicating that CAL, rather than COL, readily interacted with the O_V_ site. Even though COL may not diffuse out as soon as possible, its weak adsorption does not significantly affect the new reaction cycle. Additionally, Pt NPs@MnO_x_ exhibits considerable selectivity in the hydrogenation of different unsaturated aldehydes (Fig. 3b). For furfural and 3-methylbut-2-enal, hydrogenation of the aldehyde group is still the predominant reaction, highlighting the excellent C=O selectivity on Pt NPs@MnO_x_. Owing to the advantage of abundant O_V_ for the selective adsorption of the C=O group on the MnO_x_ shell, a unique hollow structure for reactant enrichment and controllable spatial location to optimize catalytic performance, Pt NPs@MnO_x_ enables catalysing the selective hydrogenation of CAL under lower reaction conditions than other catalysts, such as Pt–Sn nanowires, Pt_3_Co/RGO–MW, Pt/Fe_3_O_4_ and so on (Fig. 3c). Moreover, COL selectivity of >90% can be achieved over a wide range of H_2_ pressure intervals and reaction times, suggesting that it could participate in many tandem reactions with superior compatibility. Among them, a 95% conversion with 95% COL selectivity is obtained at only 0.5 MPa H_2_ and 40 min, which is a relatively mild condition compared with most reported catalytic systems. In a five-cycle stability test, stable CAL conversion and considerable COL selectivity beyond 92% are observed on the Pt NPs@MnO_x_ nanoreactor. The electronic structure and morphology features of the spent catalyst are barely altered, demonstrating the good stability of Pt NPs@MnO_x_ (Supplementary Fig. S17d and e).

According to the results of the structural characterization (Supplementary Figs S18–S20), a Pt_4_/K_2_Mn_4_O_8_ model with O_V_ is constructed to investigate the local atomic structure. The adsorbed behavior of CAL and H atoms involved in the CAL hydrogenation reaction is analysed on a Pt_4_/K_2_Mn_4_O_8_ model (Supplementary Figs S21–S23 and Supplementary Tables 2 and 4). The results show that the most stable adsorption sites for CAL and H atoms are the O_V_ site of the K_2_Mn_4_O_8_ surface and the Pt-2_Interface_ (Pt-2_I_) site, respectively. The differential charge density (side view in Fig. 3d) displays that the Pt_4_ cluster has more electron accumulation compared with K_2_Mn_4_O_8_, which results in spontaneous dissociation of H_2_. CAL adsorption configurations (Supplementary Table 2) and the differential charge density in the top view suggest that the O_V_ readily adsorbs and activates CAL. Nevertheless, for the co-adsorption of H and CAL, the distance between the H atom and CAL is too far (∼4.2 Å), leading to a failure of direct CAL hydrogenation (Supplementary Figs S24–S27). The reaction pathway should therefore be a dissociation–spillover–reaction process. As shown in Fig. 3d, H_2_ spontaneous dissociation occurring over the Pt_4_ cluster first produces adsorbed H atoms and then H atoms adsorbed at the O_Interface_ (O_I_) site spill over to the O_T_ site of the K_2_Mn_4_O_8_ surface, which is an exothermic process with the reaction energy as low as 0.44 eV. The H-atom spillover not only occurs theoretically, but also experimentally via mixing various catalysts with WO_3_ (Supplementary Fig. S28) [35–37]. In comparison with other nanoreactors with TiO_x_, CeO_x_ and SiO_2_ as shell materials, Pt NPs@MnO_x_ exhibits the best COL selectivity, as seen from Supplementary Fig. S29. The high selectivity is derived from the support. Moderate adsorption energy is the key factor (Supplementary Fig. S30). In addition, it is found that many manganese species (MnO_2_, Mn_2_O_3_ and Mn_3_O_4_) possess ideal adsorption ability for the aldehyde group when they are introduced into the catalytic system as an additive or support (Fig. 3e and Supplementary Table 5). Otherwise, DFT results also demonstrate that the aldehyde group of CAL is easily activated to generate COL and the desorption of COL is much easier than other by-products (Supplementary Table 3). Based on the above discussion, the catalytic mechanism is proposed in Fig. 3f. First, the hollow structure of the Pt NPs@MnO_x_ nanoreactor affords a confined space in which reactants enter via directional diffusion driven by the concentration gradient. Then, selective adsorption reduces the internal reactant concentrations, which would promote the in-diffusion of reactants consistently. After the reaction with abundant hydrogen atoms, the weak adsorption of products forces them to leave the nanoreactor in time.

Owing to the stronger Pt–Mn interaction [38], Pt NPs remain at high valence even under harsh hydrogen treatment conditions (Supplementary Fig. S31). If Pt NPs are metallic, competitive adsorption and strong bonding of H atoms have a limited catalytic process [39]. This phenomenon also exists on Pt NPs/Mn_3_O_4_ (Supplementary Fig. S31). It may be the reason why manganese material is rarely used in catalytic hydrogenation because the catalyst is usually reduced by H_2_ to obtain a metal phase ahead of the hydrogenation reaction. This result provides some inspiration for the design and application of manganese-containing material as the carrier for the hydrogenation reactions of oxygen-containing functional groups.

## CONCLUSION

In summary, we investigated the reactant-enrichment behaviors at the mesoscale level on Pt NPs@MnO_x_ nanoreactor catalysts. Although there are some deficiencies in the accuracy of catalyst synthesis, the improved catalytic performance on the nanoreactor is achieved by the reactant enrichment. This effect is systematically studied in the thermocatalytic process by using various characterization techniques and multiscale process engineering. For the CAL-selective hydrogenation reaction, the Pt NPs@MnO_x_ nanoreactor is one of the most efficient catalysts (good COL selectivity) to date in terms of reaction conditions. The mechanism includes diffusion, selective reactant adsorption and enhanced adsorption strength, which ultimately determine the catalytic performance of nanoreactors. Our findings offer the possibility to enhance the catalytic performance at the mesoscale level by designing a rational nanoreactor, rather than reducing the size of the metal particles or modifying them with heteroatoms or ligands at the nanoscale level.

## Supplementary Material

nwad201_Supplemental_File
