# Supplementary material for: Reactant enrichment in hollow void of Pt NPs@MnOx nanoreactors for boosting hydrogenation performance
Source: Natl Sci Rev. 2023 Jul 20;10(10):nwad201. doi: 10.1093/nsr/nwad201 (PMC10476892; doi:10.1093/nsr/nwad201)
Supplement: nwad201_Supplemental_File [file nwad201_supplemental_file.pdf]

Supporting Information for

Reactant Enrichment in Hollow Void of Pt NPs@MnO_x Nanoreactors for Boosting Hydrogenation Performance

Yanfu Ma[1], Liwei Wang[1,2], Wantong Zhao[3], Tianyi Liu[1,4], Haitao Li[1], Wenhao Luo[5,6], Qike Jiang[7], Wei Liu[7], Qihua Yang[1], Jun Huang[8], Riguang Zhang[3], Jian Liu[1,4,6,9,*], G. Q. Max Lu[10] and Can Li[1,*]

[1] State Key Laboratory of Catalysis, Dalian Institute of Chemical Physics (DICP), Chinese Academy of Sciences, Dalian 116023, China;
[2] Center of Materials Science and Optoelectronics Engineering, University of Chinese Academy of Sciences, Beijing100049, China;
[3] State Key Laboratory of Clean and Efficient Coal Utilization, Taiyuan University of Technology, Taiyuan 030024, China;
[4] DICP-Surrey Joint Centre for Future Materials, Department of Chemical and Process Engineering, University of Surrey, Guildford, GU2 7XH, U.K.
[5] CAS Key Laboratory of Science and Technology on Applied Catalysis, Dalian Institute of Chemical Physics, Chinese Academy of Sciences, Dalian, 116023, China;
[6] School of Chemistry and Chemical Engineering, Inner Mongolia University, Hohhot 010021, China;
[7] Division of Energy Research Resources, Dalian National Laboratory for Clean Energy, Dalian Institute of Chemical Physics, Chinese Academy of Sciences, Dalian 116023, China;
[8] Laboratory for Catalysis Engineering, School of Chemical and Biomolecular Engineering, Sydney Nano Institute, The University of Sydney, Sydney, 2006 Australia;
[9] Center of Materials Science and Optoelectronics Engineering, University of Chinese Academy of Sciences, Beijing 100049, China;
[10] University of Surrey, Guildford, GU2 7XH, U.K

These authors contributed equally: Yanfu Ma, Liwei Wang, Wantong Zhao

[*]Corresponding author.

Email address: jian.liu@surrey.ac.uk; jianliu@dicp.ac.cn; canli@dicp.ac.cn

Table of Contents

General Information………………………………………………….…………... S1

Reagents……………………………….………………………………………. S1

Supplementary Synthesis Methods……………………………………….....….. S1

Characterization……………………………….…………………………….…..S4

Evaluation of Catalytic Activity…...……………………………………….…….. S5

Supplementary Calculation Methods………………….………………………… S6

Supplementary Finite-Element Simulation Methods……………………………... S6

Supplementary Figs S1-S38………….....…..……………………...……………..….. S7

Supplementary Tables S1-S6…..…….……………………………………………. S37

References…………………………..…………………………………...………. S40

General information

Reagents.

3-aminophenol (99.0%), ethanol (99.7%), ammonia aqueous solution (28-30%) formaldehyde (37-40%), cerium nitrate hexahydrate (>99%), potassium permanganate (>99%), cetyltrimethylammonium bromide (99.0%), tetraethyl orthosilicate (>99%), were purchased from Sinopharm Chemical Regent Company. Manganese dioxide (>99%), trimanganese tetroxide (>99%), manganese trioxide (>99%) were purchased from Shanghai Macklin Biochemical Co., Ltd. Platinum tetraaminonitrate (≥50% Pt basis), cinnamaldehyde (>99%), cinnamyl alcohol (>99%), hydrocinnamaldehyde, hydrocinnamyl alcohol (>99%), furfural (>99%), furfuryl alcohol (>99%), tetrahydrofurfuryl alcohol (>99%), dodecane (>99%), acrolein (>99%), 3-methyl-2-butenal (98%), tetrabutyl titanate (98%) purchased from m Aladdin Chemistry Co. Ltd. Crotonaldehyde (>99%), citronellal (>99%) were purchased from J&K. Chalcone (>98%) was purchased from Shanghai yuanye Bio-Technology Co., Ltd. 2-vinylbenzaldehyde (97%) was purchased from Shanghai Acmec Biochemical Co., Ltd and all chemicals were of analytical grade. All the chemical reagents were used as received without any other purification.

Supplementary Synthesis Methods

Supplementary Table A. Summary of all samples.

Entry	Sample	Entry	Sample
1	APF	6	Pt NPs@TiO_x
2	Pt NPs/APF-C	7	Pt NPs@SiO_2
3	Pt NPs@MnO_x-n and Pt NPs/MnO_x	8	Pt NPs@CeO_x
4	Pt NPs@MnO_x with different shell thickness and diameter	9	crushed sample
5	Pt NPs/Mn_2O_3, Pt NPs/Mn_3O_4 and Pt NPs/MnO_2		

1. APF The APF supports were synthesized by hydrothermal method. Typically, 3-aminophenol (2.0 g) was dissolved in a solution containing ethanol (40 mL) and deionized water (100 mL) with stirring. Subsequently, formaldehyde solution (2.8 mL) and ammonia aqueous solution (0.5 mL) were dropped in the above solution, sequentially, and then stirred for 24 h (600 rpm) at room temperature. Then, the

yellow mixed solution was transferred to a Teflon-lined stainless reactor and heated at 100°C for 24 h. Following the hydrothermal treatment, the brown precipitate was filtered and washed with deionized water and ethanol. Finally, the material was dried at 60°C overnight and APF was obtained.

2. Pt NPs/APF-C The Pt NPs/APF-C was prepared by wet-impregnation method, followed by carbonization. In brief, platinum tetraaminonitrate (40 mg) was added into deionized water (50 mL) with stirring at room temperature. APF (1 g) was dispersed in above solution and the mixture was stirred for 24 h. Subsequently, the solution needed to be hearted at 80°C to remove water from the mix solution and then dried at 60°C overnight. Finally, the sample was carbonized in N_2 atmosphere for 2 h at 250°C with a temperature ramping rate of 5 ^{o}C/min. The as-prepared material was denoted as Pt NPs/APF-C.

3. Pt NPs@MnO$_x$-n and Pt NPs/MnO$_x$ The Pt NPs@MnO_x nanoreactor was fabricated by coating Mn species on the surface of Pt NPs/APF-C. Pt NPs/APF-C (200 mg) was dispersed in deionized water (20 mL) and sonicated for 15 min. 95.0 mg $KMnO_4$ was dissolved in deionized water (10 mL) and then dropped into above solution with vigorous stirring for 4 h at room temperature. The composite was collected by centrifugation, washed with water and dried at 60°C overnight. Subsequently, the composite was calcined at 300°C in air for different time with same temperature ramping rate (5 ^{o}C/min). The Pt NPs@MnO_x-n nanoreactor was obtained, where n referred to the calcination time. Pt nanoparticles located on the outer surface was prepared as a reference. The procedure was similar with Pt NPs@MnO_x except for operation sequence. Pt impregnation was carried out on APF which had been coated with $KMnO_4$ and dried overnight. Later, the sample was calcined at 300°C in air for 4 h with a same temperature ramping rate. The as-prepared sample was denoted as Pt NPs/MnO_x.

4. Pt NPs@MnO$_x$ with different shell thickness and diameter Pt NPs@MnO_x nanoreactors with different shell thickness were synthesized via controlling the amount (20 and 120 mg) of $KMnO_4$. The samples were calcined at 300°C in air for 4 h. The as-prepared samples were denoted as Pt NPs@MnO_x-Tn and Pt

NPs@MnO_x-Tk (Tn refers to Thin; Tk refers to Thick), respectively. Pt NPs@MnO_x nanoreactors with different diameter were prepared via controlling the ratio of water/ethanol (120/20 and 30/110 mL) in the procedure of APF synthesis, followed by coating Mn species. The samples were calcined at 300°C in air for 4 h. The as-prepared samples were denoted as Pt NPs@MnO_x-S and Pt NPs@MnO_x-L (S refers to Small; L refers to Large), respectively.

5. Pt NPs/Mn_2O_3, Pt NPs/Mn_3O_4 and Pt NPs/MnO_2 These Mn supports were purchased directly and used without any treatment. Platinum tetraaminonitrate (40 mg) was added into deionized water (50 mL) with stirring at room temperature. Additionally, Mn support (1.0 g) was dispersed in above solution and stirring for 24 h. Subsequently, the solution needed to be heated at 80°C to remove water from the mix solution and dried at 60°C overnight. Finally, the sample was calcined at 300°C in air for 2 h with a temperature ramping rate of 5 ^{o}C/min. The as-prepared materials were denoted as Pt NPs/Mn_2O_3, Pt NPs/Mn_3O_4 and Pt NPs/MnO_2, respectively.

6. Pt NPs@TiO_x Typically, 100 mg Pt NPs/APF-C was dispersed in 20 mL ethanol and sonicated for 20 min. The above solution was transferred to an ice water bath and then 0.2 mL ammonia and 7 mL acetonitrile were added into them. Later, the mixed solution of 1 mL acetonitrile and 0.4 mL tetrabutyl titanate and 3 mL ethanol was dropped into the above solution with stirring for 5 h. The composite was centrifuged and dried at 100°C overnight. Finally, the sample was calcined at 400°C for 2 h with a temperature ramping rate of 5 ^{o}C/min.

7. Pt NPs@SiO_2 Typically, 0.134 g cetyltrimethylammonium bromide, 8 mL ethanol and 0.2 mL ammonia were added into 20 mL deionized water and stirred for 0.5 h. 100 mg Pt NPs/APF-C was dispersed in them with sonication for 0.5 h. Then, 0.2 mL tetraethyl orthosilicate was dropped into the above solution following with stirring for 24 h. The composite was centrifuged and dried at 60°C overnight. Finally, this sample was calcined at 500°C for 2 h with a temperature ramping rate of 5 ^{o}C/min.

8. Pt NPs@CeO_x Typically, 100 mg Pt NPs/APF-C was dispersed in 30 mL ethanol and sonicated for 15 min, followed by dropping the mixed solution of 30 mL deionized water dissolving 130 mg $Ce(NO_3)_3$ $6H_2O$ and 130 mg

hexamethylenetetramine with vigorous stirring for 2 h at room temperature. Then the mixture was heated in an oil bath at 75°C for 4 h. The composite was filtered, washed several times with deionized water and dried at 60°C overnight. Finally, the composite was calcined at 300°C in air for 4 h with a temperature ramping rate of 5 ^{o}C/min. The Pt NPs/CeO_x nanoreactor was obtained.

9. crushed sample We use liquid nitrogen to freeze the sample and then grind it for 30 min to break the hollow structure[1]. Symbol "&" is used to mark the broken samples.

Characterization

Transmission electron microscopy (TEM) images were obtained from a Hitachi HT7700. The scanning electron microscopy (SEM) images were obtained from Quanta 200F and Hitachi S5500, respectively. The high-angle annular dark fields scanning transmission electron microscopy (HAADF-STEM) and energy-dispersive X-ray spectroscopy (EDX) elemental mapping were carried on JEM-ARM200F (JEOL, Japan) at the acceleration voltage of 200 kV (Dalian Institute of Chemical Physics). The particle size distribution of Pt nanoparticles was determined by Nano Measurer 1.2 software.

The content of Pt was collected by inductively coupled plasma optical emission spectroscopy (ICP-OES, ICPS-8100, Shimadzu). X-ray diffraction (XRD) patterns were recorded on a Rigaku D/Max2500PC diffractometer with Cu Kα radiation (λ = 1.5418 Å) at room temperature in the 2θ range from 5 to 75° (5° min^{-1}). Thermogravimetric Analysis (TGA) was measured with a PerkinElmer TGA4000 using nitrogen or air as a carrier gas. X-ray photoelectron spectroscopy (XPS) was determined by a Thermofisher Excalab X+ spectrometer equipped with a monochromated aluminum source (Al Kα= 1486.6 eV). The binding energies were corrected with C 1s of 284.80 eV.

The Fourier transform infrared spectroscopy (FTIR) spectra were collected with a Nicolet 6800 spectrometer equipped with a MCT-A detector cooled by liquid nitrogen. A high pressure/high temperature cell from Specac capped with ZnSe windows was used for *in situ* measurements. 25 mg sample was pressed into

self-supported discs of approximately 13 mm in diameter and placed in a sample holder at the centre of the cell. CAL vapor was introduced into the cell by N_2 at room temperature. The spectra were collected by recording 32 scans at a resolution of 6 cm^{-1}.

Electron spin resonance spectroscopy (EPR) measurements was carried out on a Brucker EPR A200 spectrometer (microwave frequency, 9.82 GHz; sweep width, 100 G; modulation frequency, 200 kHz). Nitrogen adsorption-desorption was conducted on a Micromeritics Tristar II 2460 automated analyzer at 77 K. Before analysis, the samples need to be degassed at 120 ^{o}C under vacuum overnight. The specific surface area was calculated using the Brunauer-Emmett-Teller (BET) method. UV-vis spectra were collected using a UV-vis Spectrophotometer (Shimadzu UV-2700). Spectra was collected from 250 to 400 nm. The samples were first soaked in 1.5 mL of ethanol for 12 h. Subsequently, 1.5 mL of ethanol solution of cinnamaldehyde (30 mg/L) was added and left to adsorb for 6 h before testing.

Evaluation of catalytic activity

The selective hydrogenation of CAL was performed in a 50 mL stainless steel autoclave equipped with a magnetic stirrer. In a typical reaction, the mixtures of CAL (10 mg), catalyst (10 mg), dodecane (5 mg) and ethanol (1.5 mL) were added into the autoclave and flushed with H_2 for 4 times to replace the inner air. Dodecane is the internal standard for quantification. The stainless-steel autoclave was pressured with 2 MPa H_2. Afterward, it was heated to 70°C. After finishing the reaction, the autoclave was cooled to room temperature. For normal pressure reactions at 70°C, the hydrogenation reactions were carried out in a 25 mL round-bottom flask with refluxing and 50 mL/min H_2. The liquid products were analyzed by gas chromatograph (Shimadzu 2030) with an SH-STABLIWAX-DA capillary column (30 m × 0.25 mm × 0.25 μm), a flame ionization detector (FID), and an auto-injector. For the reusability and stability, the solution was centrifuged and then fresh CAL, dodecane and ethanol was added into the autoclave for the next cycling reaction.

Supplementary Calculation methods

Spin-polarized density functional theory (DFT) calculations were performed using the Vienna Ab initio Simulation Package (VASP) plane-wave DFT code[2]. The electronic exchange-correlation functions were described using the generalized gradient approximation (GGA)[3] with the Perdew-Burke-Ernzerhof (PBE) functional[4]. A kinetic energy cutoff of 400 eV was used to expand the electronic wave functions. The projector-augmented plane wave (PAW) was used to describe the electron-ion interactions[5, 6]. A 3×3×1 *k*-point grid of surface Brillouin-zone was employed. The optimization convergence accuracy was less than 0.08 eV $Å^{-1}$ and 1×10^{-5} eV for the force and energy.

Supplementary Finite-Element Simulation Methods.

The finite-element analysis (FEA) simulation was conducted using COMSOL Multiphysics 5.5. To reduce the computational cost, the numerical simulation was implemented in a 2D mode. The model of Pt NPs@MnO_x and Pt NPs&MnO_x were plotted as spherical shell and arched shell with inner radius of 750 nm and thickness of 30 nm, respectively. Microchannels with widths of 10 nm were plotted to denote the porous structure of samples. This model could effectively simplify the structure of samples while maintaining the mass-transfer property of mesopores. A square region with a side length of 6000 nm was filled with water, which served as the calculated domain. Due to the low Reynolds number regime (Re $\sim 10^{-4}$) resulting from the small size of samples, the "Laminar Flow" module was selected to simulate the flow field. In the stationary simulation, the left boundary was set to be an inlet with an inflow velocity of 0.235 m s^{-1} to model the flow induced by the stir. The right boundary was set to be an outlet with pressure equal to zero. All other boundaries were set to be nonslip boundaries. The flow field was solved by the Navier–Stokes equations:

$$\nabla\cdot u=0$$

$$\rho(u\cdot\nabla)u=\nabla\cdot[-pI+\mu(\nabla u)+(\nabla u)^T]+F$$

Where *u* is the velocity, ρ is the density, *p* is the pressure, and *F* is the volume force vector.

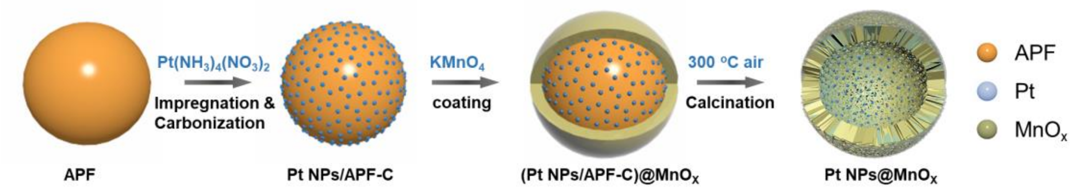

Supplementary Fig S1 | Synthetic route of Pt NPs@MnO_x nanoreactor.

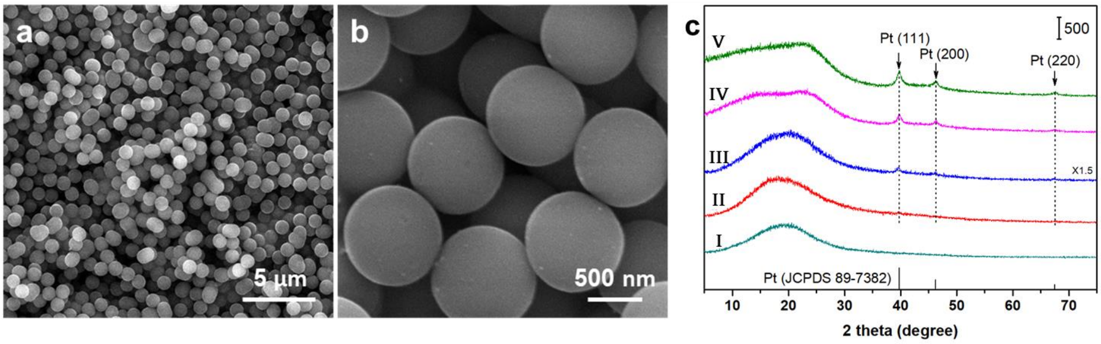

Supplementary Fig S2 | a,**b**, Scanning electron microscopy (SEM) images of APF. **c**, XRD patterns of as-prepared APF (I), and Pt NPs/APF calcinating at 120, 250, 375 and 500°C (II-V).

As shown in Supplementary Figs S2a,b, as-prepared APF is homogeneous sphere. After Pt loading, the sample was carbonized at 120, 250, 375 and 500°C, respectively. The corresponding XRD pattern result is displayed in Supplementary Fig S2c. When the carbonization temperature is 250°C, Pt diffraction peaks appeared and the density increases with the increase of temperature. To avoid the agglomeration of Pt particles, a low temperature carbonization treatment was selected at N_2 atmosphere for 2 h at 250°C.

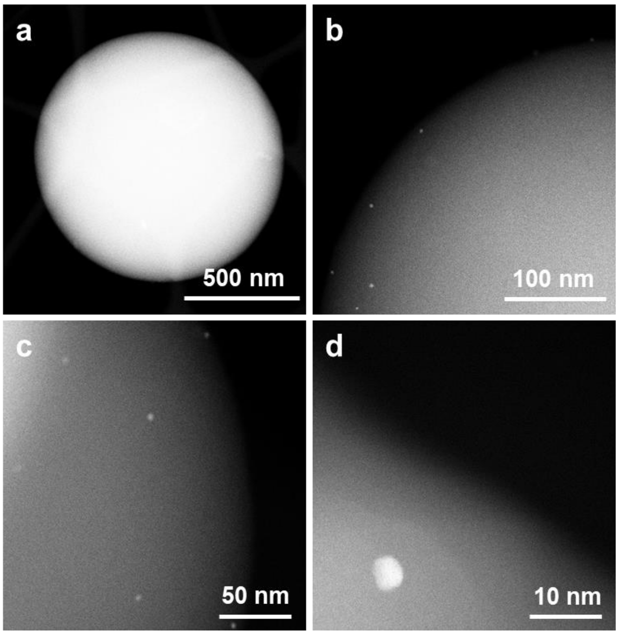

Supplementary Fig S3 | a-d, Aberration-corrected high-angle annular dark-field scanning transmission electron microscopy (HAADF-STEM) images of Pt NPs/APF-C at low and high magnifications.

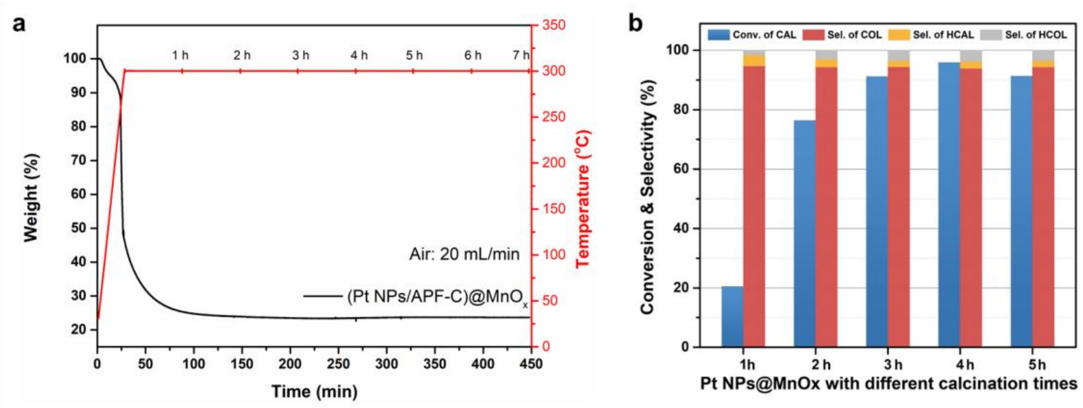

Supplementary Fig S4 | a, The thermal gravimetric analysis curves of (Pt NPs/APF-C)@MnO_x at 300°C for 7 h. **b**, Conversion of CAL and the product distribution over Pt NPs@MnO_x with different calcination times at 70°C, 15 min and 2 MPa H_2.

According to the thermal gravimetric result, when the temperature reaches 300°C, APF decomposes greatly (Supplementary Fig S4a). The catalytic performances were evaluated over Pt NPs@MnO_x nanoreactor with different calcination times for the selectivity hydrogenation of CAL to COL as shown in Supplementary Fig S4b. During the process of catalyst calcination, except for the continuous decomposition of

APF, the shell thickness is changed, which may affect the catalytic activity. It is noteworthy that although the conversions are different, the selectivity does not alter substantially from Supplementary Fig S4b, suggesting that the CAL adsorbs on the shells in the same manner as it enters the hollow void. Thus, the rate of CAL molecular diffusion into the interior of the nanoreactor determines the reaction conversion. In Supplementary Fig S5c, it is observed that shell thickness of Pt NPs@MnO_x-1 is 66.7 nm, which is larger than than of Pt NPs@MnO_x-2, so that the rate of reactant diffusion is limited. Therefore, the weaker enrichment by occupation of the hollow void and the greater diffusion resistance result in a less active for Pt NPs@MnO_x-1. A volcanic curve of conversion was observed. The CAL conversion increased from 20 to 95% as the calcination time increasing from 1 to 4 h and then decreased slightly. Calcination for 4 hours is the optimal condition.

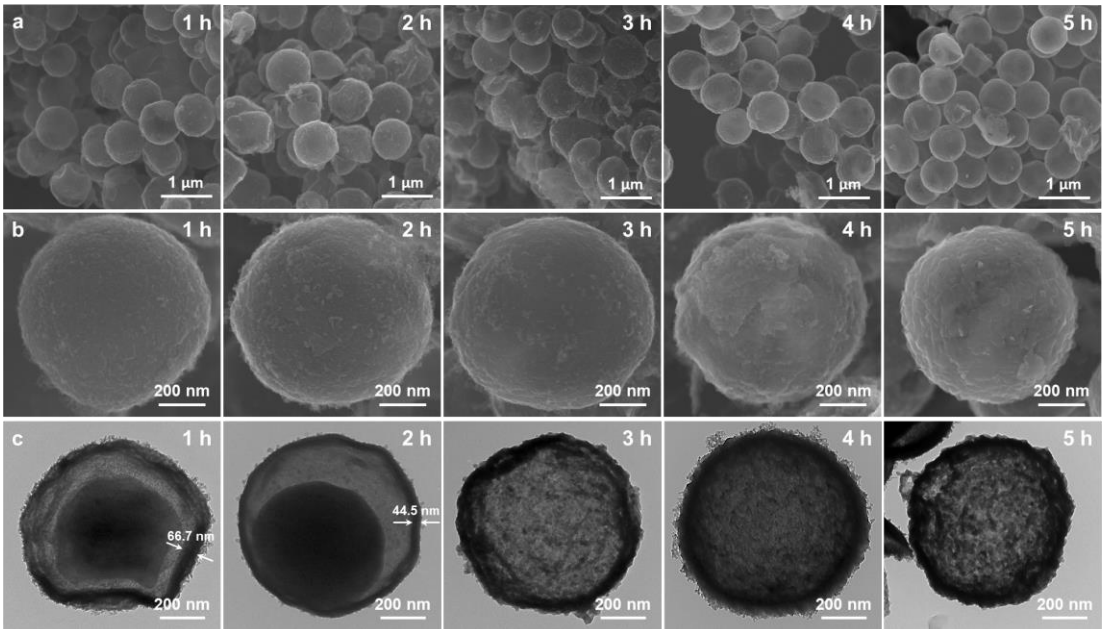

Supplementary Fig S5 | SEM images (**a,b**) and transmission electron microscopy (TEM) images (**c**) of Pt NPs@MnO_x-n with different calcination time.

TEM images of Pt NPs@MnO_x-n with different calcination time show that APF disappeared after a calcination time of 3 h in air (Supplementary Fig S5c). The final sample is calcinated for 4 h, guaranteeing the removal of APF. In HAADF-STEM

measurement which can detect much higher resolution internal structural information. As shown in Supplementary Fig S10b, there is no residual carbon fringes around Pt NPs lattices on MnO_x substrate. In Supplementary Fig S18a, XRD patterns indicate that the final Pt NPs@MnO_x is composed of Pt and Mn species, free of carbon species. In addition, thermogravimetric analysis with an extended time at 300°C is also conducted for (Pt NPs/APF-C)@MnO_x, which contains the intact carbonized APF. Strikingly, no obvious weight loss can be observed as the temperature maintains at 300°C from 3 to 7 h, demonstrating the complete removal of the organic polymer APF. Those results could prove that Pt NPs@MnO_x contain no apparent carbon after being calcinated at 300°C for 4 h.

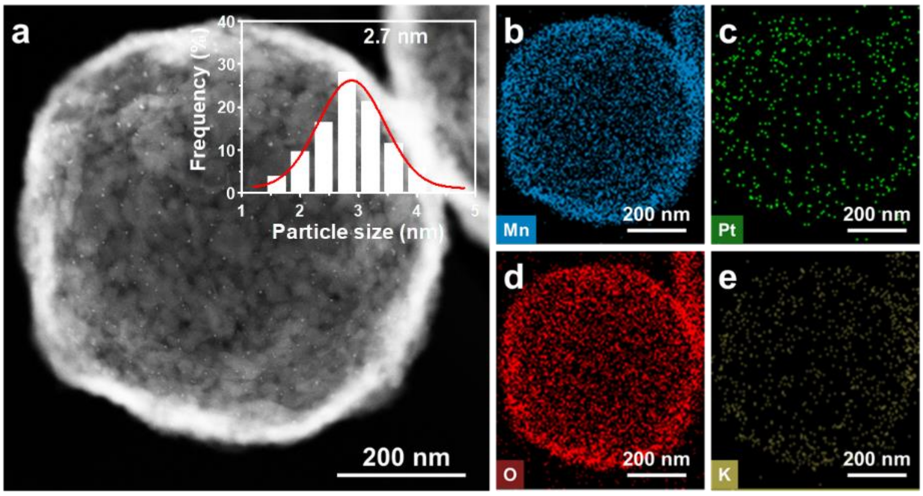

Supplementary Fig S6 | a, Aberration-corrected HAADF-STEM image of Pt NPs@MnO_x-3. The inset in **a** shows the corresponding size histograms of Pt NPs. **b-e**, The corresponding energy-dispersive X-ray spectroscopy elemental mapping images of Mn, Pt, O and K.

The particle size can be affected by many factors, such as calcination time/temperature/atmosphere, support material, the rate of heating, etc. In this work, the particle size change slightly under different treatment times. Although the Pt particle size varies from 2.7 to 2.3 nm with an increase in calcination time, no apparent changes in catalytic performance in terms of activity (91-96%) and CAL selectivity (~94%) can be observed for catalysts with different Pt particle sizes, as shown in Supplementary Fig S4b. This suggests that the variation of the Pt particle size doesn't greatly alter the catalytic performance.

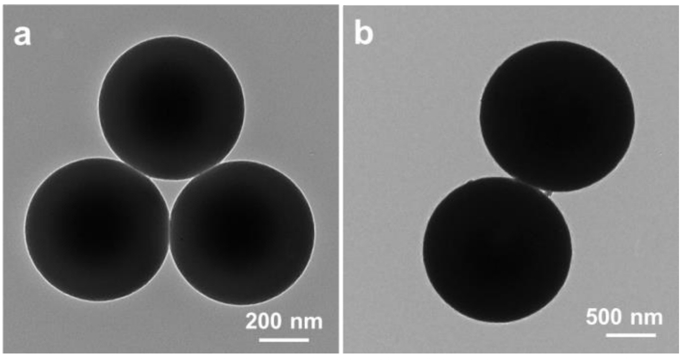

Supplementary Fig S7 | a, TEM image of APF-Small. **b**, TEM image of APF-Large.

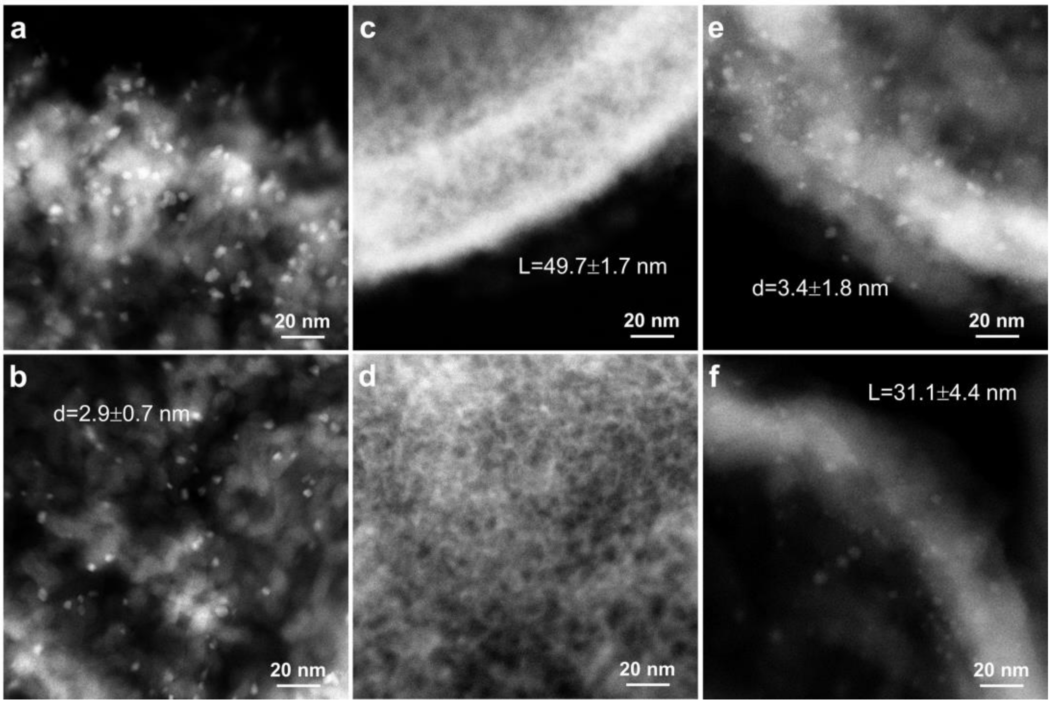

Supplementary Fig S8 | HAADF-STEM images of Pt NPs@MnO_x-Tn (**a, b**) Pt NPs@MnO_x-Tk (**c, d**) and Pt NPs/MnO_x (**e, f**).

Interestingly, the thickness of hollow MnO_x shell shows notable impacts on the distribution of Pt NPs. It can be observed that Pt NPs are randomly scattered on MnO_x skeleton in Pt NPs@MnO_x-Tn, where Pt NPs are completely shielded by wholly thick MnO_x shell in Pt NPs@MnO_x-Tk. Pt NPs migration might be associated with serious shrinkage and wrinkle of MnO_x shell during thermal decomposition of thin $KMnO_4$ layers, whereas the surface layer but not the skeleton MnO_x exist slight variation during thermal decomposition of thick $KMnO_4$ layers. Besides, Pt NPs/MnOx are also prepared as a reference to demonstrate the reactant enrichment effect.

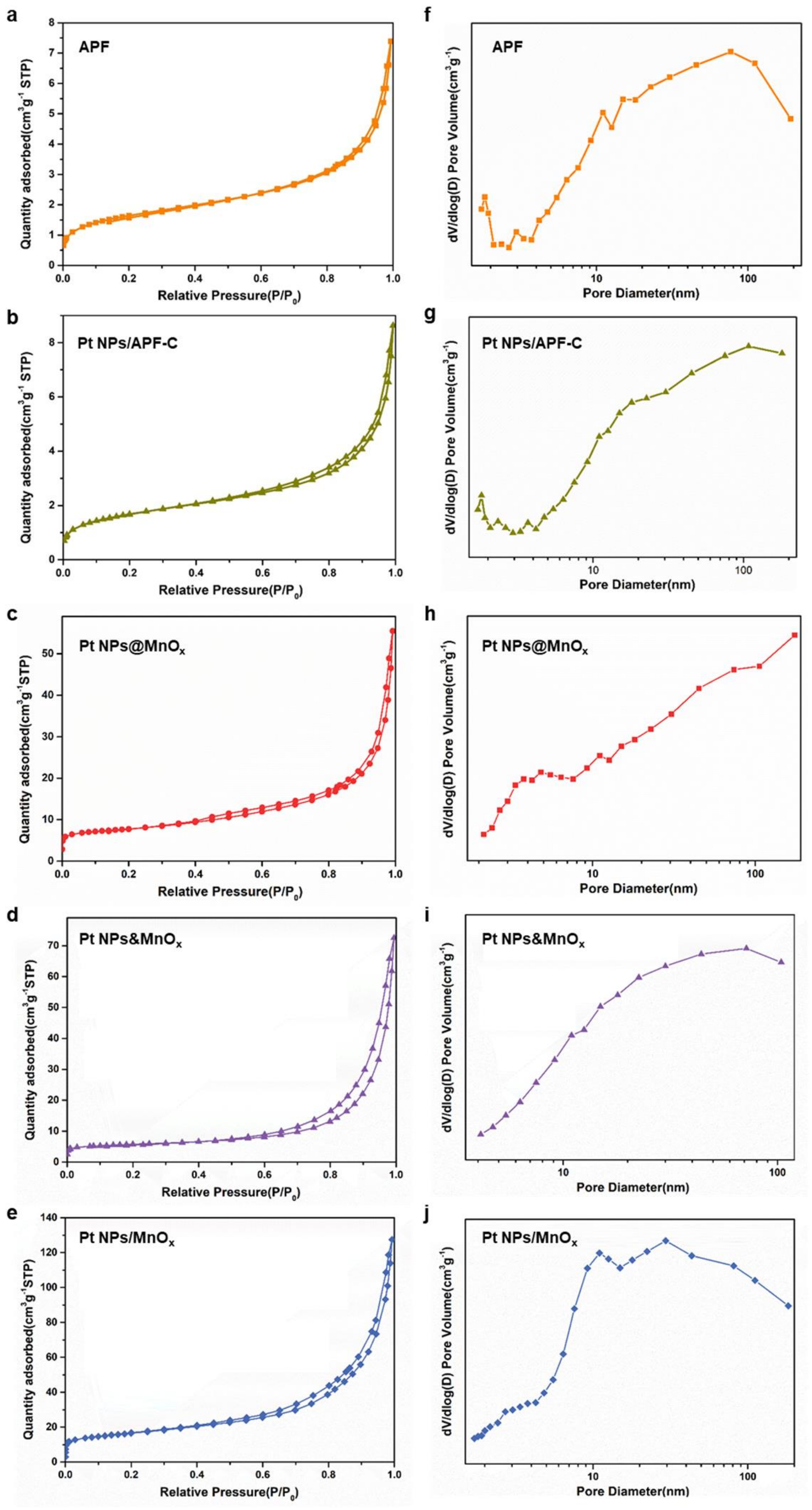

Supplementary Fig S9 | a-e N_2 adsorption-desorption isotherms of and **f-j**, The corresponding pore-size distribution curves of APF, Pt NPs/APF-C, Pt NPs@MnO_x, Pt NPs&MnO_x, and Pt NPs/MnO_x, respectively.

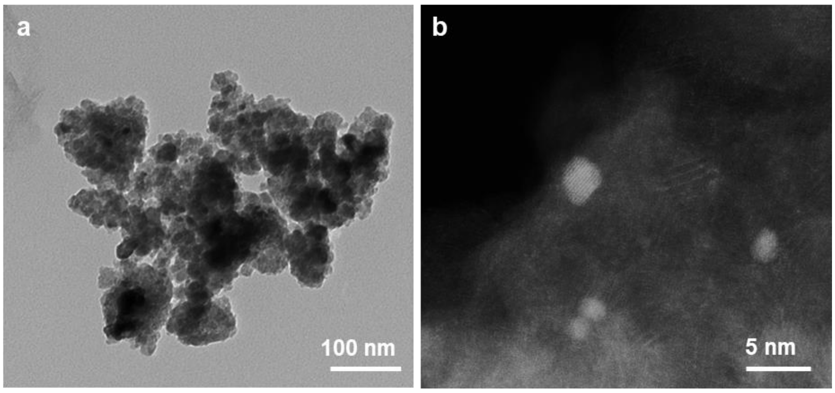

Supplementary Fig S10 | a,b, TEM image (a) and HAADF-STEM image (b) of Pt NPs&MnO_x.

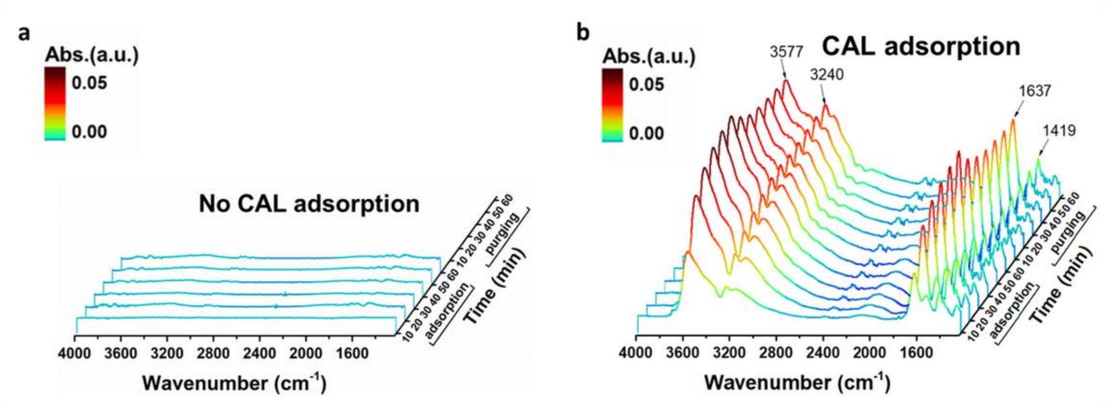

Supplementary Fig S11 | *In situ* FTIR spectras using CAL over Pt NPs/APF-C (a) and Pt NPs@MnO_x (b) after exposure to CAL vapor and subsequent N_2 purging at room temperature.

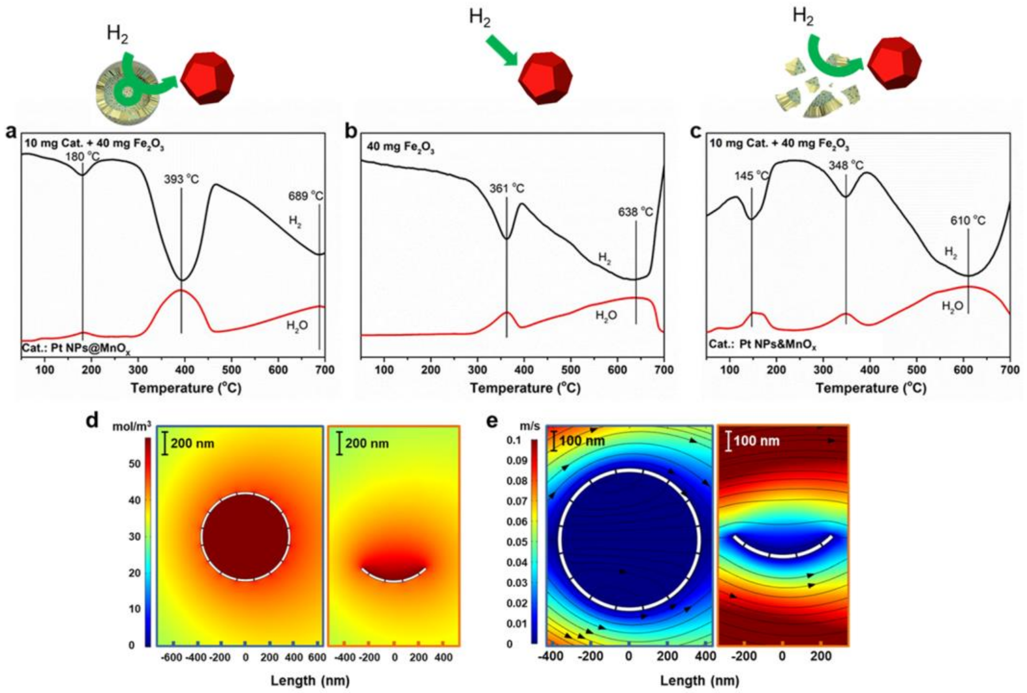

Supplementary Fig S12 | a-c, H_2-TPR-MS curves of physical mixtures of Fe_2O_3 with Pt NPs@MnO_x, Fe_2O_3, and physical mixtures of Fe_2O_3 and Pt NPs&MnO_x, respectively. Red line is assigned to H_2O signal. Black line is assigned to H_2 signal. **d,** The simulated concentration profiles of dissociated H from H_2 in Pt NPs@MnO_x (left) and Pt NPs&MnO_x (right) elucidated by the finite-element simulation. **e**, The simulated velocity field of Pt NPs@MnO_x (left) and Pt NPs&MnO_x (right) elucidated by the finite-element simulation.

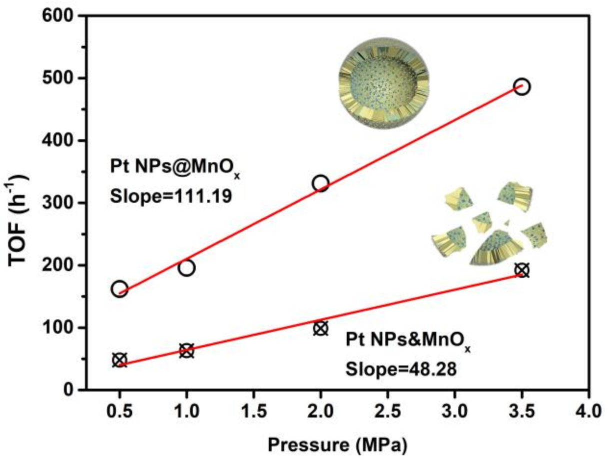

Supplementary Fig S13 | Activity comparison of Pt NPs@MnO_x and Pt NPs&MnO_x at 70°C, 10 min and various H_2 pressure.

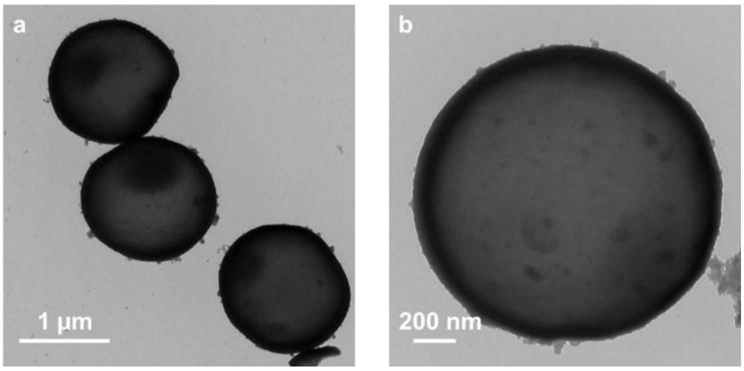

Supplementary Fig S14 | TEM images of Pt NPs@MnO$_x$ with the size of ~1400 nm in diameter and a shell thickness of ~32 nm by calcination for 6 h.

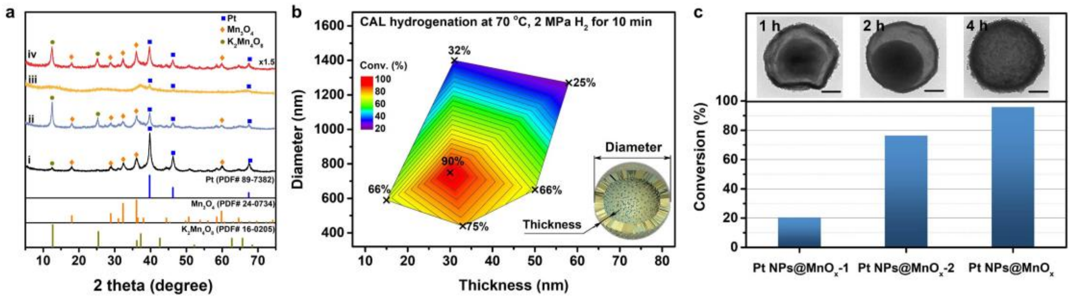

Supplementary Fig S15 | a, XRD patterns of Pt NPs@MnO$_x$-Tn (i), Pt NPs@MnO$_x$ (ii), Pt NPs@MnO$_x$-Tk (iii)and Pt NPs/MnO$_x$ (iv), respectively. **b**, Effect of nanoreactor structural parameters on catalytic activity. **c**, Effect of residual APF on activity after occupying cavity. The scale bar is 200 nm.

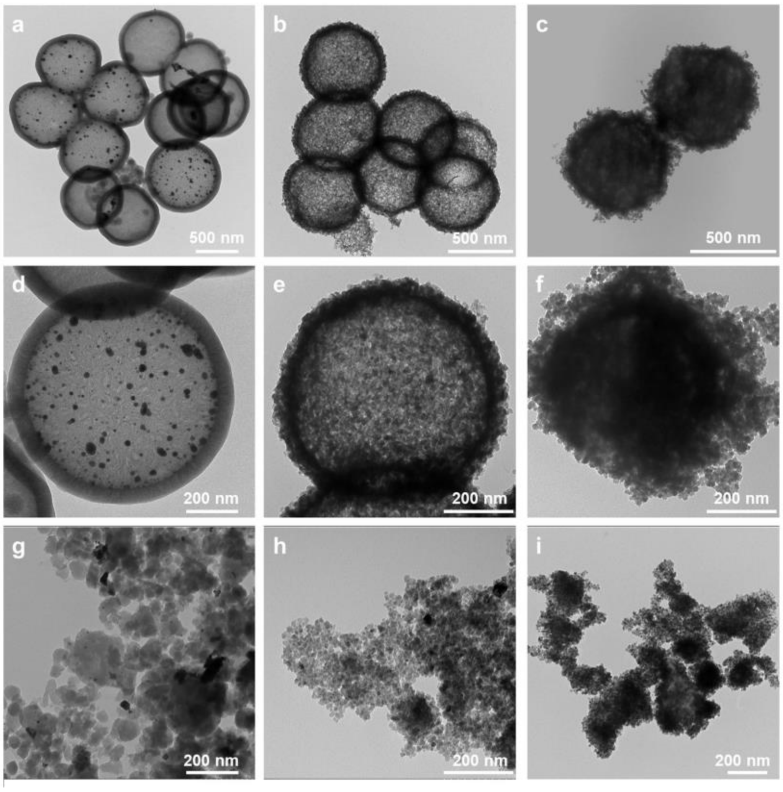

Supplementary Fig S16 | a,d, TEM images of Pt NPs@SiO_2. **b,e**, TEM images of Pt NPs@TiO_2. **c,f**, TEM images of Pt NPs@CeO_2. **g-i**, TEM images of corresponding crushed samples.

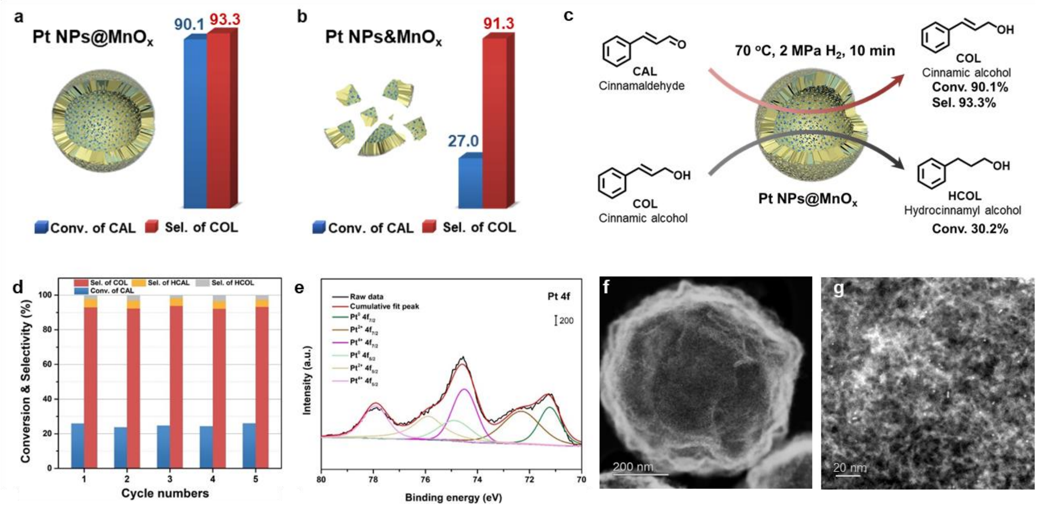

Supplementary Fig S17 | a,b, Performance comparison over Pt NPs@MnO_x (a) and Pt NPs&MnO_x (b) nanoreactor with different shell at 70°C, 10 min and 2 MPa H_2. **c**, Performance comparison by using CAL and COL as substrates, respectively. **d**, Cycling performance of Pt NPs@MnO_x for CAL selective hydrogenation at 30°C, 25 min and 0.5 MPa H_2. **e**, X-ray photoelectron spectroscopy (XPS) of Pt 4f level in Pt NPs@MnO_x after reaction at 70°C, 15 min and 2 MPa H_2. It is crushed before XPS measurement. **f,g**, HAADF-STEM image of used Pt NPs@MnOx at 70°C, 15 min, and 2 MPa H_2.

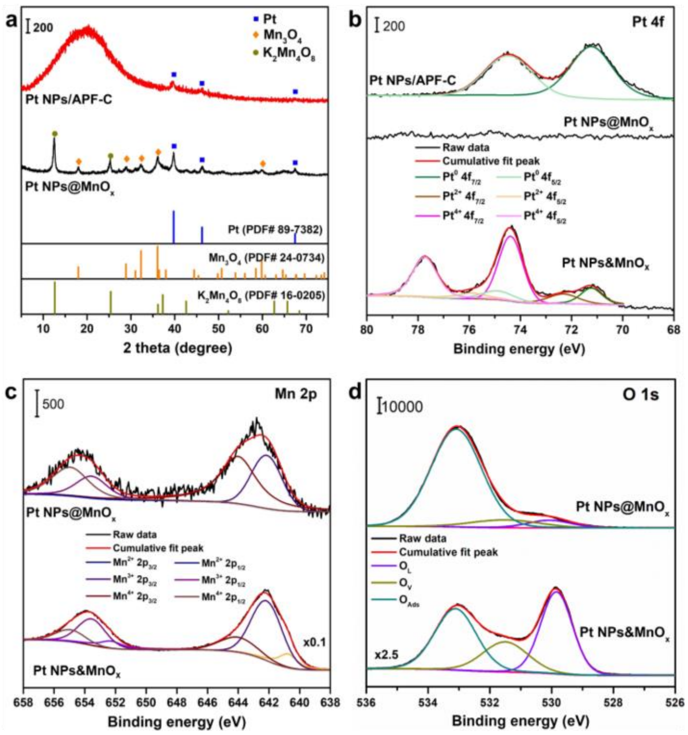

Supplementary Fig S18 | a, X-ray diffraction patterns of Pt NPs/APF-C and Pt NPs@MnO_x. **b**, XPS profiles of Pt 4f level in Pt NPs/APF-C, Pt NPs@MnO_x and Pt NPs&MnO_x. **c,d**, XPS profiles of Mn 2p, O 1s level in Pt NPs@MnO_x and Pt NPs&MnO_x.

We find that the crystal phase of MnO_x shell contains a mixture of Mn_3O_4 and $K_2Mn_4O_8$ in Pt NPs@MnO_x according to XRD patterns (Supplementary Figs S18a, S19a). Pt 4f spectra shows the metallic Pt is converted into oxidized state on the inner surface of shell owing to the strong oxidizability, which could be characterized over crushed nanoreactor by grinding because the shell thickness exceeds the probe depth resulting in internal Pt NPs failing to be detected in X-ray photoelectron spectroscopy (XPS) experiment over Pt NPs@MnO_x (Supplementary Figs S18b, S19b). Additionally, XPS profiles of Mn 2p level over Pt NPs&MnO_x could reflect the binding energy of interior Mn species. Comparing with Pt NPs@MnO_x in Supplementary Fig S18c, the peak position shifted to lower binding energy with the appearance of Mn^{2+} and the increase of Mn^{3+}. The formation of partially low-valent Mn on the internal surface originates from the occurrence of electron transfer from Pt to the proximate Mn.

It has been reported that the exposure of Mn^{3+} can give rise to surface O_V

formation to maintain electrostatic balance.[7, 8] In our work, XPS measurement was carried out on Pt NPs@MnO_x and Pt NPs&MnO_x to prove the existence of Ov. Mn $2p_{3/2}$ could be divided into three peaks after peak fitting and deconvolution, corresponding to Mn^{2+}, Mn^{3+} and Mn^{4+}. In Supplementary Fig S18c, Mn^{3+} could be found in both Pt NPs@MnO_x and Pt NPs&MnO_x, suggesting the existence of O_V. Additionally, the peak of O_V at 531.5 eV were also found from O 1s region in Supplementary Fig S18d. We also perform the EPR measurement to detect O_V. The existence of O_V is further confirmed by the observation of apparently symmetric peaks at g≈2.003 in EPR spectra over Pt NPs&MnO_x, as shown in Supplementary Fig S20.

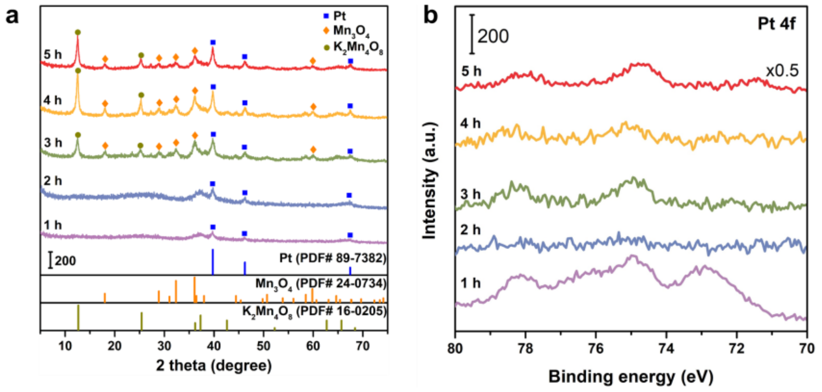

Supplementary Fig S19 | XRD patterns (a) and XPS profiles (b) of Pt NPs@MnO_x with different calcination times.

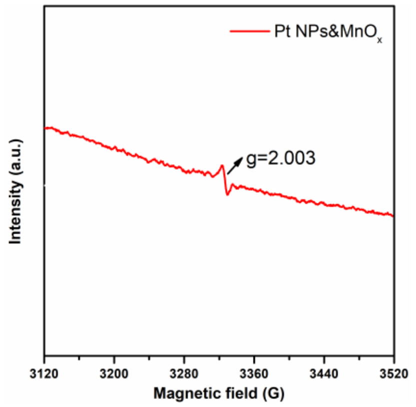

Supplementary Fig S20 | Electron paramagnetic resonance (EPR) spectrum of Pt NPs&MnO_x.

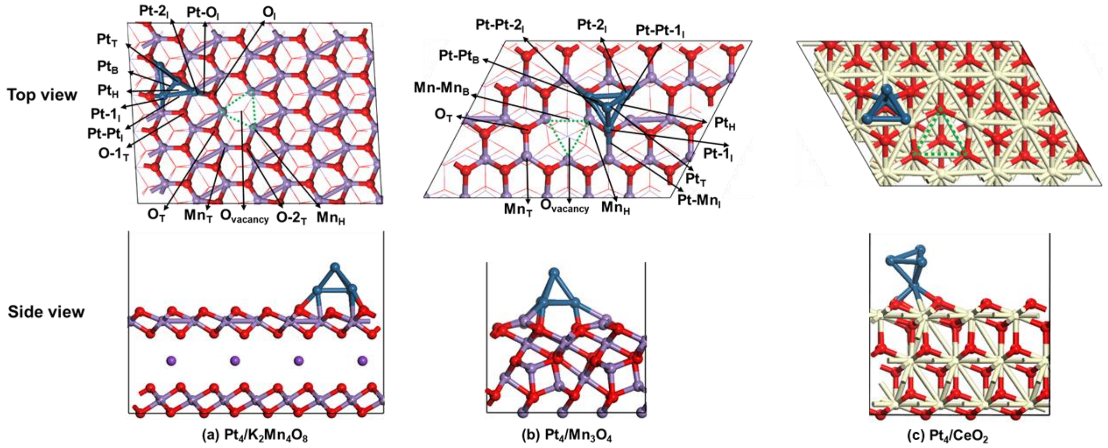

Supplementary Fig S21 | The optimized structure and the possible adsorption sites of (**a**) $Pt_4/K_2Mn_4O_8$, (**b**) Pt_4/Mn_3O_4 and (**c**) Pt_4/CeO_2 catalysts with the top and side views, the green triangle represents O_V. The purple, dark purple, red, blue, and yellow balls represent Mn, K, O, Pt and Ce atoms, respectively. T represents the top site; I represents the interface site; H represents the hollow site; B represents the bridge site.

According to experimentally proved surface structure of Pt NPs@MnO_x, the MnO_x shell consists mainly of $K_2Mn_4O_8$, and the Pt NPs are firmly anchored in the internal surface of hollow structure. Aiming at analyzing the underlying factor that Pt NPs@MnO_x catalyst performs excellent catalytic performance toward cinnamaldehyde (CAL) selective hydrogenation compared with Pt NPs/Mn_3O_4 catalyst, the $Pt_4/K_2Mn_4O_8$ and Pt_4/Mn_3O_4 catalysts are constructed. Since the generation of oxygen-vacancy (O_V) over the Mn_3O_4 and $K_2Mn_4O_8$ surface easily occurs[9, 10], $Pt_4/K_2Mn_4O_8$ catalyst is constructed by Pt_4 cluster supported on $K_2Mn_4O_8$ with the O_V, in which $K_2Mn_4O_8$ surface is modeled by a three-layer $p(5\times4)$ supercell; the O and Mn atoms of the top layer together with the adsorbates are relax during the calculations. Meanwhile, Pt_4/Mn_3O_4 catalyst is constructed by Pt_4 cluster supported on Mn_3O_4 with the O_V, in which Mn_3O_4 surface is modeled using a nine-layer $p(3\times2)$ supercell, the top six layers and the adsorbates are relaxed. As the model of Pt NPs@CeO_2, Pt_4/CeO_2 catalyst is constructed by Pt_4 cluster supported on CeO_2 with the O_V, in which CeO_2 surface is modeled using a nine-layer $p(4\times3)$ supercell, the top five layers and the adsorbates are relaxed.

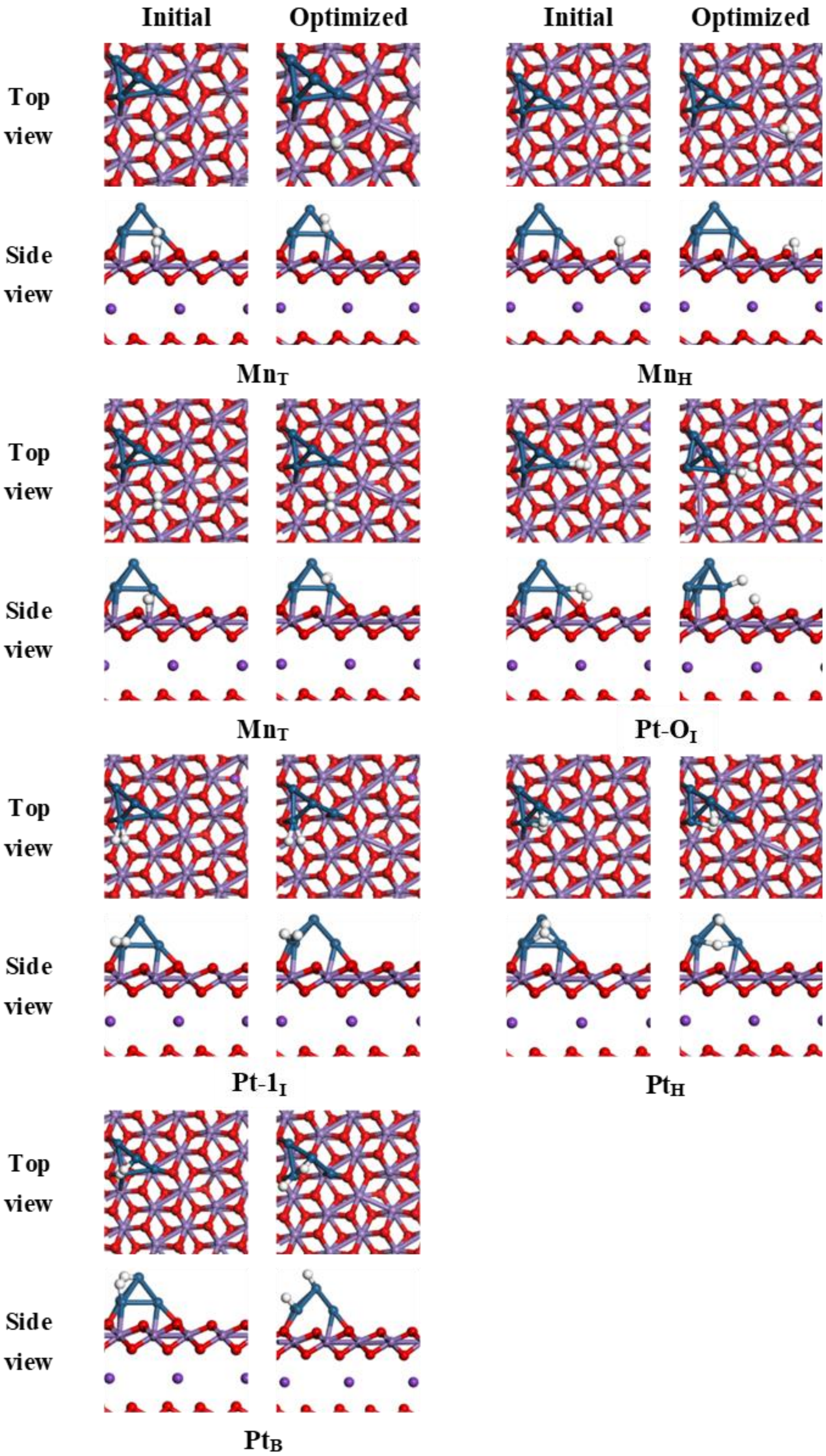

Supplementary Fig S22 | The initial and optimized structures of H_2 adsorbed at different sites on $Pt_4/K_2Mn_4O_8$ catalyst.

Since H_2 dissociation is the key initial step to provide hydrogen source for CAL selective hydrogenation, H_2 adsorption and dissociation were firstly investigated at

different adsorption sites over the $Pt_4/K_2Mn_4O_8$ and Pt_4/Mn_3O_4 catalysts. Specifically, for $Pt_4/K_2Mn_4O_8$ catalyst, as shown in Supplementary Fig S22, our results show that H_2 initially adsorbed at the Pt_4 cluster (Pt_H site), the interface Pt site between Pt_4 cluster and $K_2Mn_4O_8$ (Pt-1_I, Pt-O_I and Pt_B sites) takes place the spontaneously dissociative adsorption to form the adsorbed H atoms, whereas H_2 initially adsorbed over $K_2Mn_4O_8$ surface (Mn_T and Mn_H sites) prefers the molecular adsorption. These results show that the spontaneously dissociative adsorption of H_2 into H atom all occurs at the Pt cluster and the interface Pt site in $Pt_4/K_2Mn_4O_8$ catalyst, namely, the Pt cluster can provide abundant hydrogen source to participate into the hydrogenation reaction.

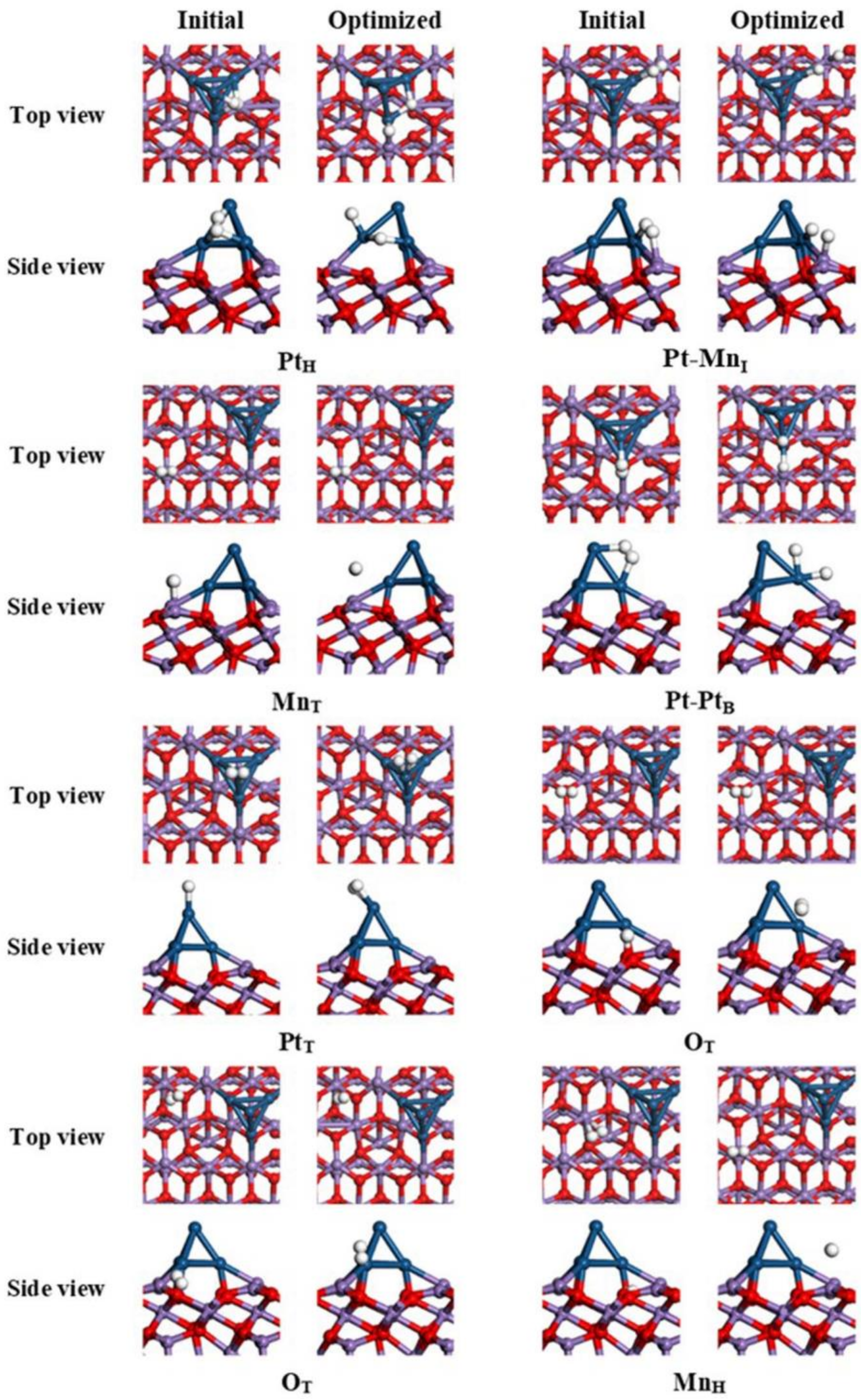

Supplementary Fig S23 | The initial and optimized structures of H_2 adsorbed at different sites on Pt_4/Mn_3O_4 catalyst.

For Pt_4/Mn_3O_4 catalyst, as shown in Supplementary Fig S23, the Pt_4 cluster (Pt_T and Pt_H sites) and the interface Pt sites between Pt_4 cluster and Mn_3O_4 surface (Pt-Mn_I and Pt-Pt_B sites) also prefer H_2 spontaneous dissociation to form adsorbed H atoms, whereas the molecular adsorption occurs over Mn_3O_4 surface (Mn_T, Mn_H and O_T sites)

instead of the dissociative adsorption. Thus, the Pt_4 cluster of Pt_4/Mn_3O_4 catalyst can also provide abundant hydrogen source for CAL selective hydrogenation, which is well supported by the previously reported results[11, 12], for example, Wu *et al.*[11] found that Pt nanoparticles is preferable for H_2 dissociation to H atoms on Pt/TiO_xH_y catalyst instead of TiO_xH_y surface, subsequently, H atoms spillover to TiO_xH_y surface to participate into CAL hydrogenation reaction. Wei *et al.*[12] also reported that the Pt is responsible for H_2 dissociation on Pt/$CeZrO_2$ catalyst in CAL hydrogenation reaction.

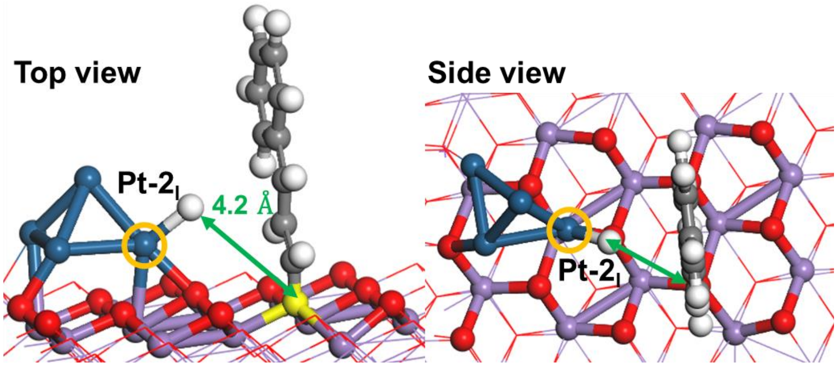

Supplementary Fig S24 | The distance between the adsorption site of CAL and H atom when H atom is adsorbed on the Pt-2_I site. Yellow atom is the O atom of CAL; The orange circle represents Pt-2_I site.

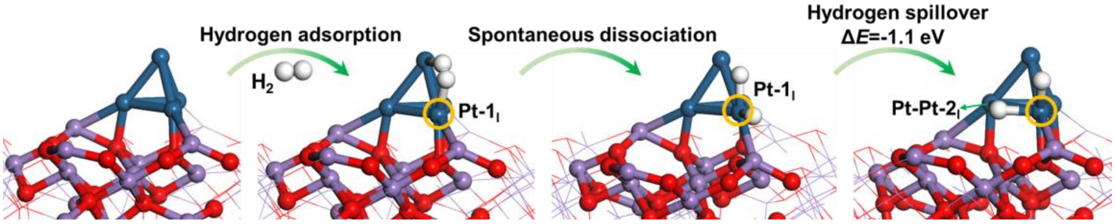

Supplementary Fig S25 | H atom spillover on Pt_4/Mn_3O_4 catalyst. The orange circle represents Pt-1_I site.

Further, the adsorbed and co-adsorbed behavior of H and CAL involved in CAL hydrogenation are analyzed on Pt_4/Mn_3O_4 catalyst (Supplementary Table S2, S4), the results show that the most stable adsorption site of CAL is the O_V site of Mn_3O_4 surface; H atom prefers to be adsorbed at the interface Pt sites between Pt_4 cluster and Mn_3O_4 surface (Pt-Pt-1_I, Pt-Pt-2_I and Pt-2_I). Thus, as shown in Supplementary Fig S25, H_2 spontaneous dissociation occurred over Pt_4 cluster firstly produces adsorbed

H atoms, and then H atom adsorbed at the Pt-1_I site spillover to the Pt-Pt-2_I site, which is a strongly exothermic process with the reaction energy of 1.1 eV. Thus, the H atom spillover occurs from the Pt-1_I site to Pt-Pt-2_I site, then, both H adsorbed at Pt-Pt-2_I site and CAL adsorbed at the O_V site participate into CAL hydrogenation reaction.

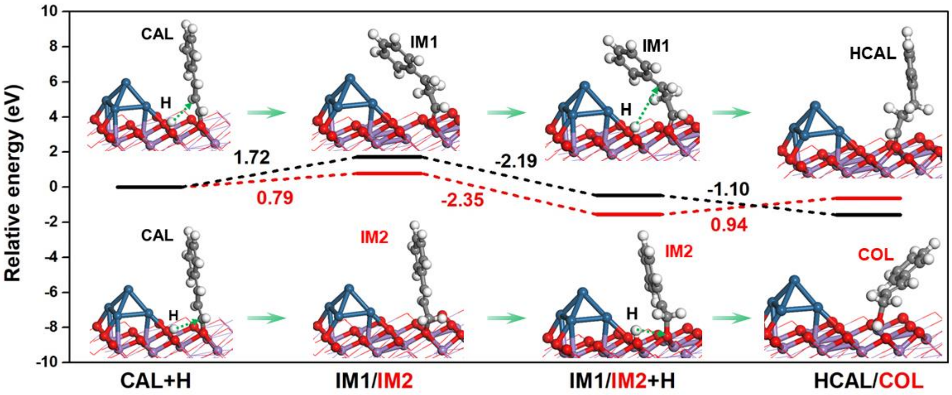

Supplementary Fig S26 | Potential energy profile for HCAL and COL production from CAL hydrogenation on $Pt_4/K_2Mn_4O_8$ catalyst together with the corresponding structures.

Above DFT adsorption calculations demonstrated that CAL is readily interacting with O_V, aiming to further analyze the preference of HCAL and COL generation from CAL hydrogenation on $Pt_4/K_2Mn_4O_8$, the reaction energies of HCAL and COL generation pathways are calculated. As shown in Supplementary Fig S26, for HCAL generation, the pathway of CAL+H→ IM1, IM1→IM1+H and IM1+H→HCAL require the reaction energies of 1.72, -2.19, and -1.10 eV, respectively, suggesting that the step of CAL+H→IM1 is strongly endothermic, and it is the rate-determining step. DFT studies by Dai *et al.*[13] also found that the rate-determining step is CAL+H→IM1 in the process of CAL hydrogenation to HCAL on PtFe catalyst. However, for COL generation, the pathway of CAL+H→IM2, IM2→IM2+H and IM2+H→COL require the reaction energies of 0.79, -2.35 and 0.94 eV, respectively, suggesting that the step of IM2+H→COL is the rate-determining step, which is much lower than the

rate-determining step of HCAL generation (0.94 *versus* 1.72 eV). Based on above analysis, it is concluded that COL generation is more energetically favorable than HCAL generation from CAL hydrogenation on $Pt_4/K_2Mn_4O_8$ catalyst, namely, the aldehyde group of CAL is easily activated at the O_V site to facilitate the hydrogenation of aldehyde group to generate COL, which is consistent with the experimental results. Interestingly, the adsorption energy of COL is much lower than that of HCAL (-1.21 *versus* -1.83 eV) (Supplementary Table S3), which means that the desorption of COL is much easier than that of HCAL on $Pt_4/K_2Mn_4O_8$ catalyst. Moreover, Wu *et al.*[11] experimentally and theoretically found that CAL prefers to be adsorbed at the O_V site of TiO_xH_y surface via O atom on Pt/TiO_xH_y catalyst leading to the adsorption of CAL with its C=O bond preferentially than C=C bond, which is advantageous for CAL hydrogenation to COL. Wei *et al.*[12] also reported that the carbonyl group of CAL strongly interacts with O_V on $Pt/CeZrO_2$ catalyst, which results in the easy activation of its C=O bond to perform excellent activity and selectivity for COL generation in CAL hydrogenation reaction.

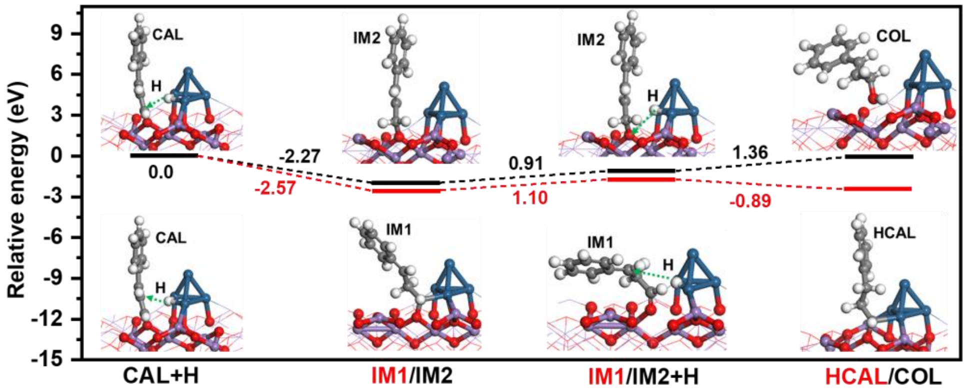

Supplementary Fig S27 | Potential energy profile for HCAL and COL production from CAL hydrogenation on Pt_4/Mn_3O_4 catalyst together with the corresponding structures.

As shown in Supplementary Fig S27, for HCAL generation, the path of CAL+H→IM1, IM1→IM1+H and IM1+H→HCAL require the reaction energies of -2.57, 1.10 and -0.89 eV, respectively, suggesting that the step of IM1→IM1+H is strongly endothermic, and it is the rate-determining step. However, for COL

generation, the path of CAL+H→IM2, IM2→IM2+H and IM2+H→COL require the reaction energies of -2.27, 0.91 and 1.36 eV, respectively, suggesting that the step of IM2+H→COL is the rate-determining step, which is higher than the rate-determining step for HCAL generation (1.36 *vs.* 1.10 eV). As a result, HCAL generation is more energetically favorable than COL generation from CAL selective hydrogenation on Pt_4/Mn_3O_4 catalyst, namely, the C=C bond of CAL is easily activated at the O_V site to facilitate the hydrogenation of C=C bond to generate HCAL.

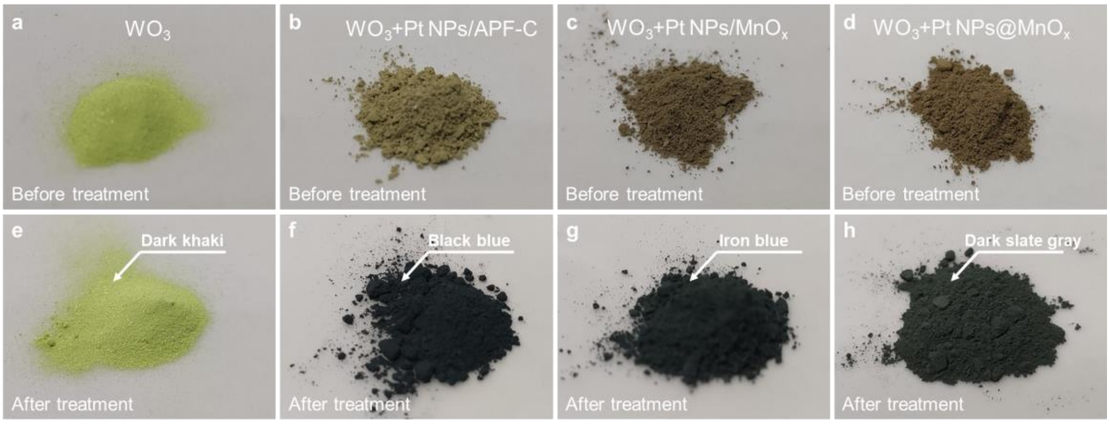

Supplementary Fig S28 | Photographs of hydrogen spillover of 3 g WO_3 (**a,e**) mixed without/with 30 mg Pt NPs/APF-C (**b,f**), Pt NPs/MnO_x (**c,g**) and Pt NPs@MnO_x (**d,h**) before/after treatment with 1 MPa H_2 at 30°C for 10 min.

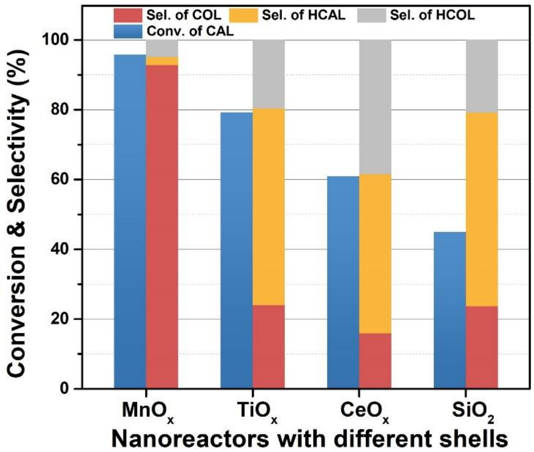

Supplementary Fig S29 | Performance comparison over nanoreactor with different shell. Reaction condition is same with that of Figure 3h.

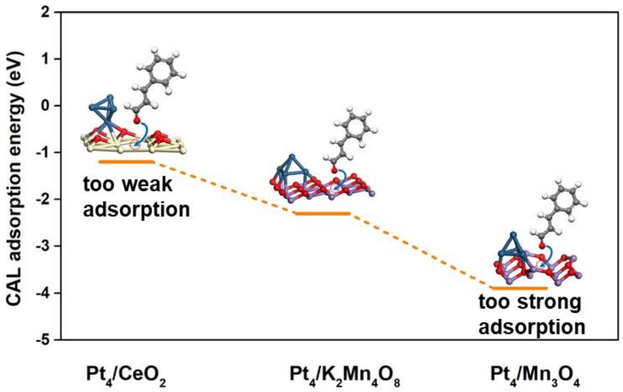

Supplementary Fig S30 | CAL adsorption energy on Pt_4/CeO_2, $Pt_4/K_2Mn_4O_8$ and Pt_4/Mn_3O_4

Taking Pt NPs@CeO_x as an example, a simplified Pt_4/CeO_2 model has been constructed to investigate CAL adsorption behavior on O_V site. In Supplementary Fig S30 and Table S2, DFT calculations reveal the weak CAL adsorption energy (-1.17 eV) on Pt_4/CeO_2, which is responsible for low COL selectivity and activity. By contrast, the strong CAL adsorption energy (-3.89 eV) on Pt_4/Mn_3O_4 model causes low conversion on Pt NPs/Mn_3O_4. Moderate adsorption energy (-2.27 eV) observed on $Pt_4/K_2Mn_4O_8$ promotes both the selectivity and activity.

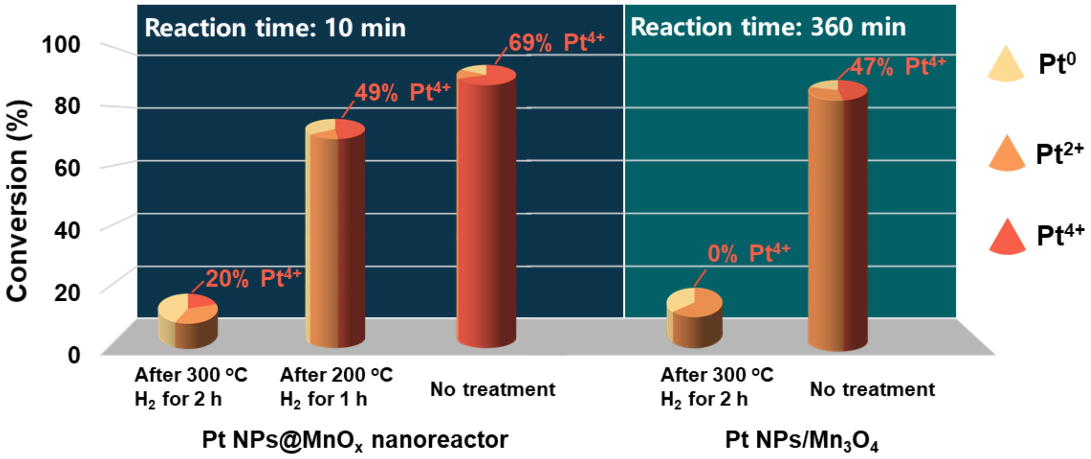

Supplementary Fig S31 | Effect of the Pt valence state on activity of CAL selectivity hydrogenation over different catalysts at 70°C and 2 MPa H_2.

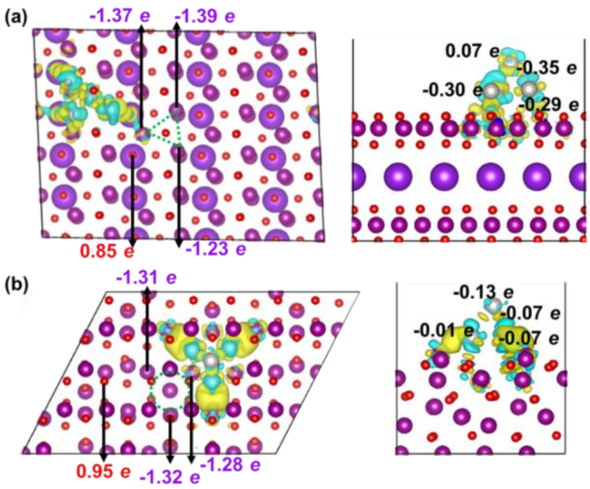

Supplementary Fig S32 | Differential charge density of surface Pt atoms on the (**a**) $Pt_4/K_2Mn_4O_8$ and (**b**) Pt_4/Mn_3O_4 catalysts. The yellow and blue shaded regions represent the charge gain and charge loss, respectively. The Bader charge of Pt atoms, O atoms and Mn atoms around the vacancy is presented, the purple is Mn atom, the red is O atom, and the grey is Pt atom.

To clarify the role of the O_V of $Pt_4/K_2Mn_4O_8$ and Pt_4/Mn_3O_4 catalysts in affecting the catalytic performance of CAL selective hydrogenation, the electronic properties of $Pt_4/K_2Mn_4O_8$ and Pt_4/Mn_3O_4 catalysts are calculated. As presented in Supplementary Fig S32, over the $Pt_4/K_2Mn_4O_8$ and Pt_4/Mn_3O_4 catalysts, the differential charge density shows that Pt_4 cluster has more electron accumulation compared with $K_2Mn_4O_8$ and Mn_3O_4 surfaces, which results in the easy occurrence of H_2 spontaneous dissociation on Pt_4 cluster. Furthermore, the Bader charge (*e*) of Pt atoms and Mn atoms around the O_V are calculated to quantitatively analyze the lose electrons number of Pt atoms and Mn atoms. As shown in Supplementary Fig S32, Mn atoms around the O_V lose more electrons than the O and Pt atoms on $Pt_4/K_2Mn_4O_8$ catalyst, and the similar situation also occurs on Pt_4/Mn_3O_4 catalyst, which leads to CAL adsorbed on O_V site.

Further, the loss in electrons number of Mn atoms around the O_V is -1.37 e, -1.39 e and -1.23 e on $Pt_4/K_2Mn_4O_8$ catalyst; the lose electrons number of Mn atoms around the O_V is -1.31 e, -1.32 e and -1.28 e on Pt_4/Mn_3O_4 catalyst. Compared to Pt_4/Mn_3O_4 catalyst, the Mn atoms around the O_V lose more electrons on $Pt_4/K_2Mn_4O_8$ catalyst, which is preferable for the C=O bond activation of CAL to generate COL rather than the C=C bond activation of CAL to generate HCAL.

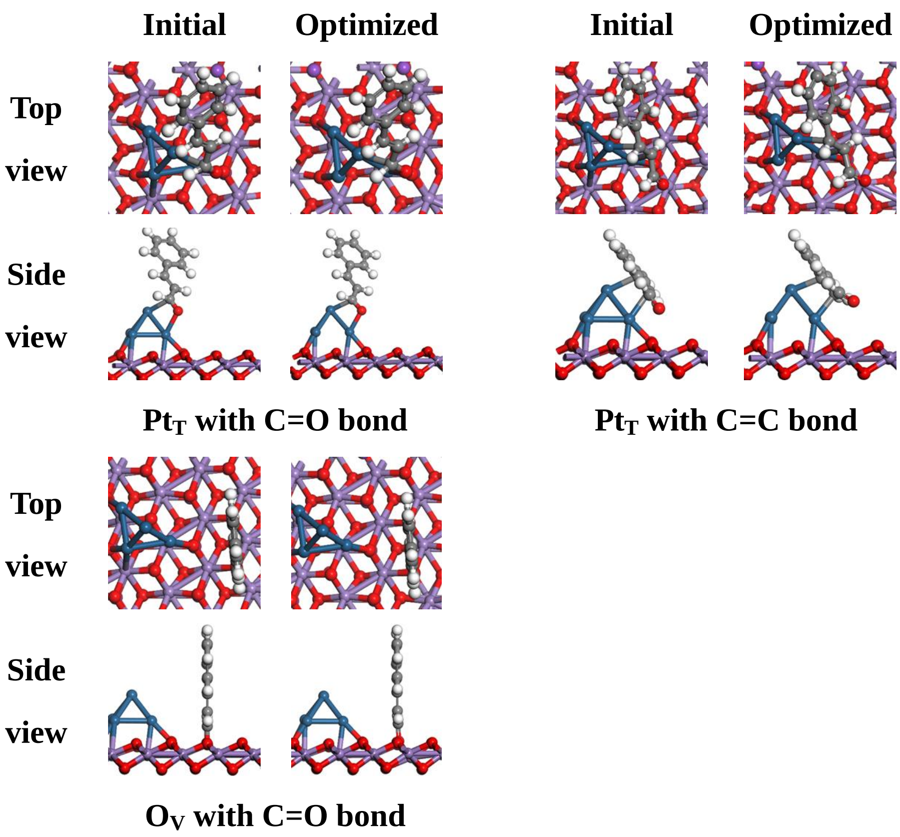

Supplementary Fig S33 | The initial and optimized structures of CAL adsorbed at different sites on $Pt_4/K_2Mn_4O_8$ catalyst.

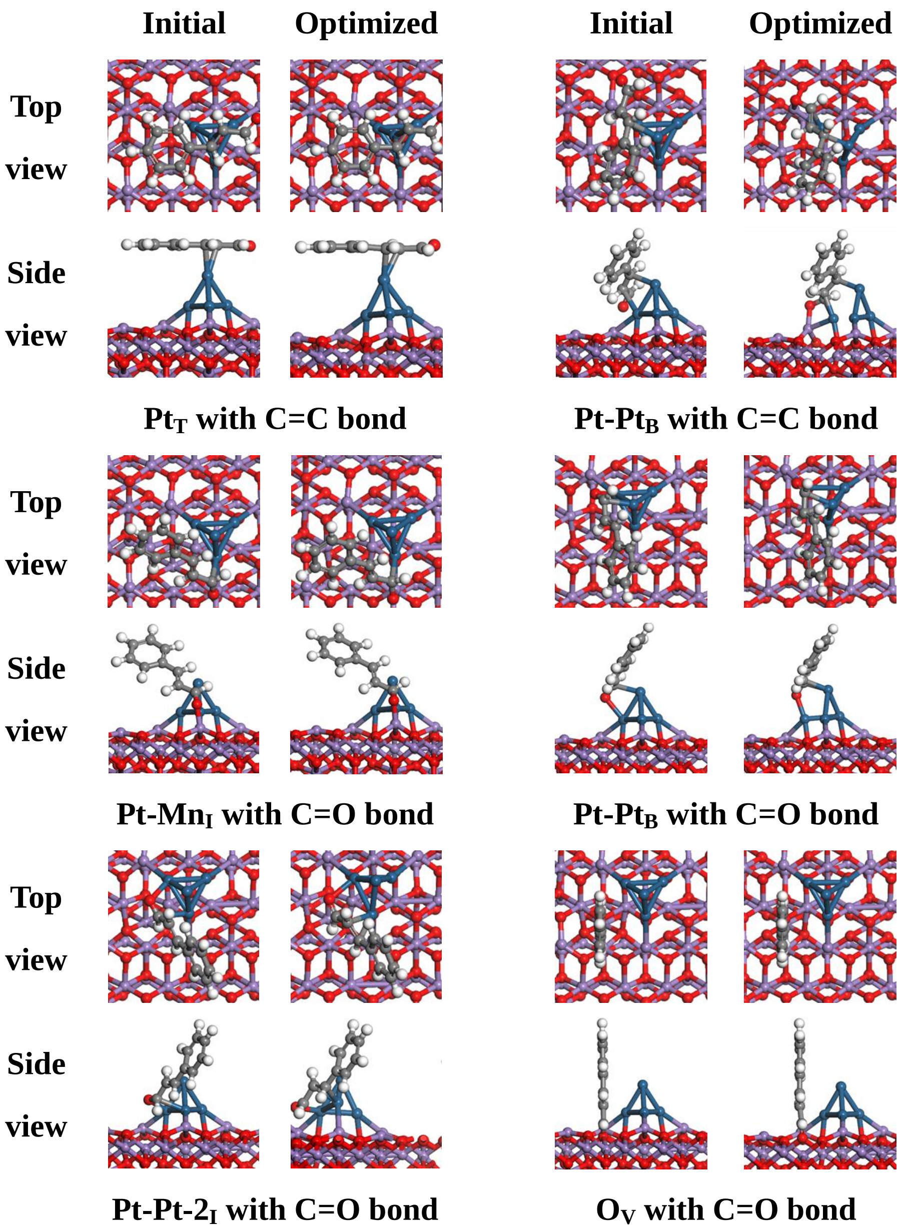

Supplementary Fig S34 | The initial and optimized structures of CAL adsorbed at different sites on Pt_4/Mn_3O_4 catalyst.

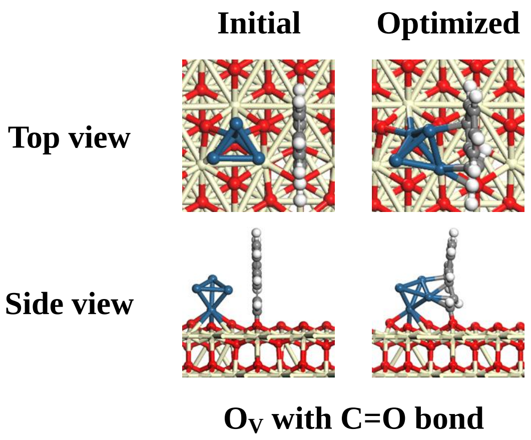

Supplementary Fig S35 | The initial and optimized structures of CAL adsorbed at different sites on Pt_4/CeO_2 catalyst.

The adsorption behavior of CAL is analyzed on $Pt_4/K_2Mn_4O_8$ catalyst, in which the different adsorption sites of $Pt_4/K_2Mn_4O_8$ catalyst are examined. As listed in Supplementary Table S2, CAL is stably adsorbed at the O_V site of $K_2Mn_4O_8$ surface via O atom with the adsorption energy of -2.27 eV; CAL can be stably absorbed at the Pt_T site of Pt_4 cluster via C=C bond with the adsorption energy of -1.67 eV; CAL can be also adsorbed at the Pt_T site of Pt_4 cluster via C=O bond with the adsorption energy of -1.28 eV. Thus, CAL adsorbed at the O_V site of $K_2Mn_4O_8$ surface is the most stable configuration.

The adsorption behavior of CAL is analyzed on Pt_4/Mn_3O_4 catalyst, in which the different adsorption sites of Pt_4/Mn_3O_4 catalyst are examined. As listed in Supplementary Table S2, CAL is stably absorbed at the O_V site of Mn_3O_4 surface via O atom with the adsorption energy of -3.89 eV; CAL can be stably absorbed at the Pt_T site of Pt_4 cluster via C=C bond with the adsorption energy of -2.19 eV; CAL can be stably absorbed at the Pt-Pt_B site of Pt_4 cluster via C=C bond with the adsorption energy of -2.59 eV; CAL can be adsorbed at the Pt-Mn_I site of Pt_4 cluster via C=O bond with the adsorption energy of -2.34 eV; CAL can be adsorbed at the Pt-Pt_B site of Pt_4 cluster via C=O bond with the adsorption energy of -3.47 eV; CAL can be adsorbed at the Pt-Pt-2_I site of Pt_4 cluster via C=O bond with the adsorption energy of

Initial Optimized Initial Optimized

-2.32 eV. Thus, CAL adsorbed at the O_V site of Mn_3O_4 surface is the most stable configuration.

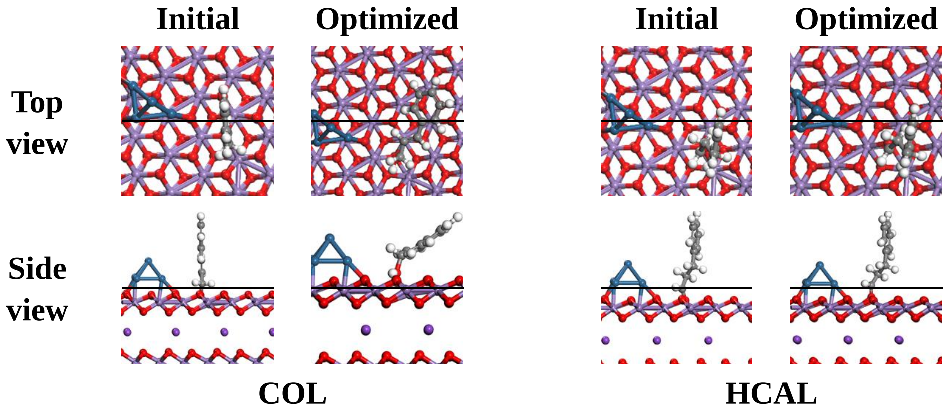

Supplementary Fig S36 | The initial and optimized structures of COL and HCAL adsorbed at O_V site on $Pt_4/K_2Mn_4O_8$ catalyst.

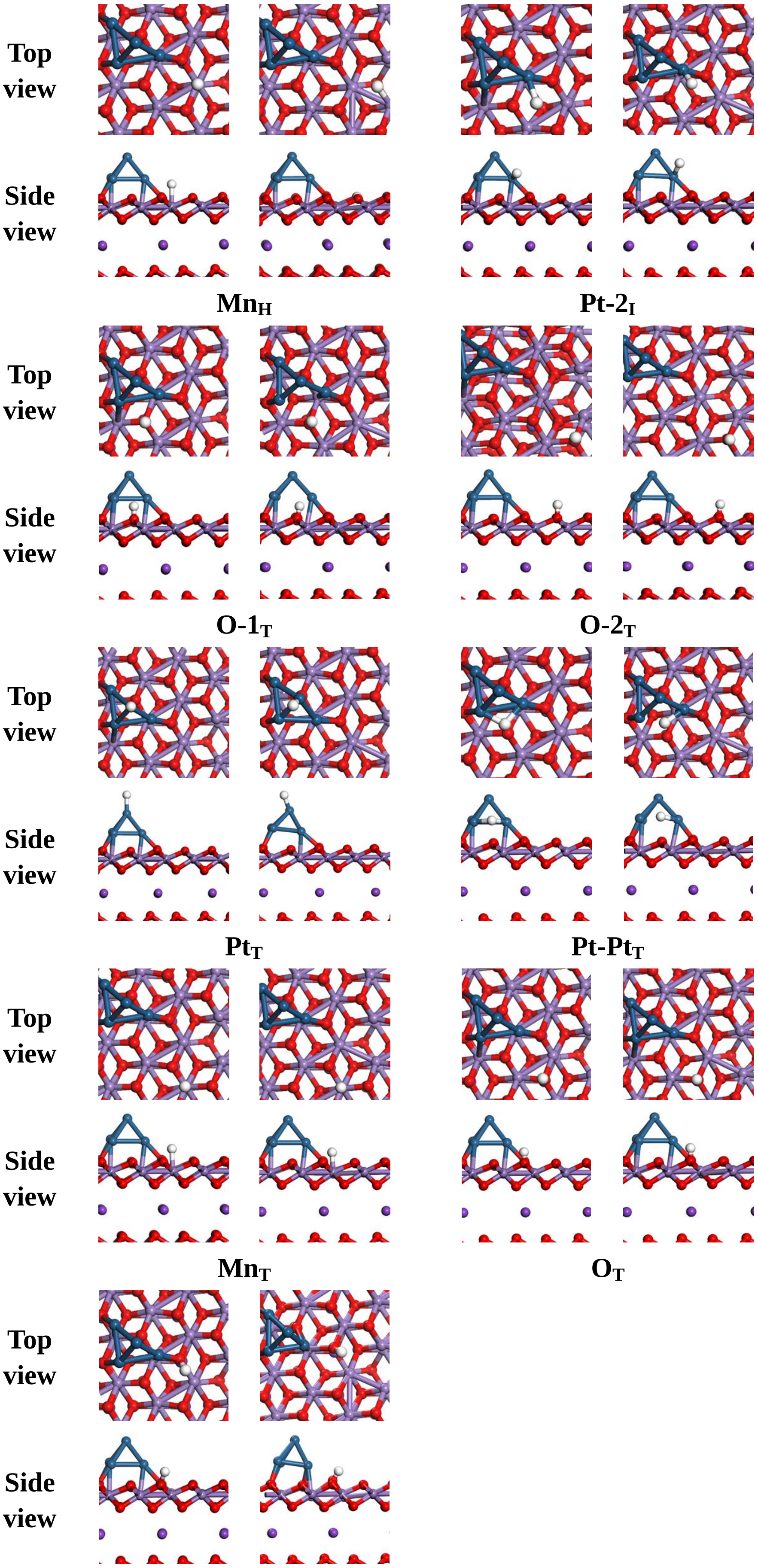
Top view
Side view
Mn_H
$Pt\text{-}2_I$
$O\text{-}1_T$
$O\text{-}2_T$
Pt_T
$Pt\text{-}Pt_T$
Mn_T
O_T

O_I

Supplementary Fig S37 | The initial and optimized structures of H adsorbed at different sites on $Pt_4/K_2Mn_4O_8$ catalyst.

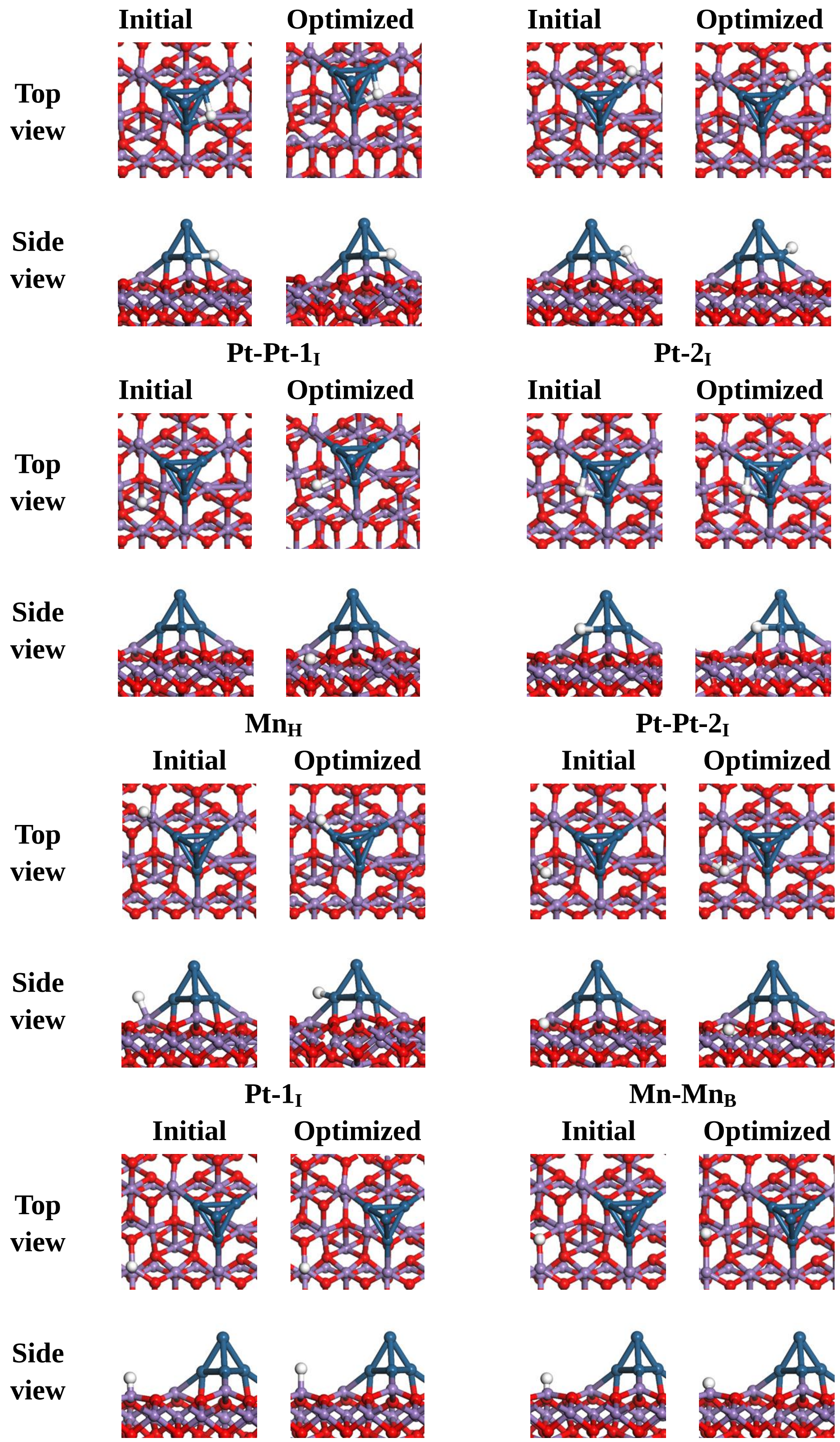

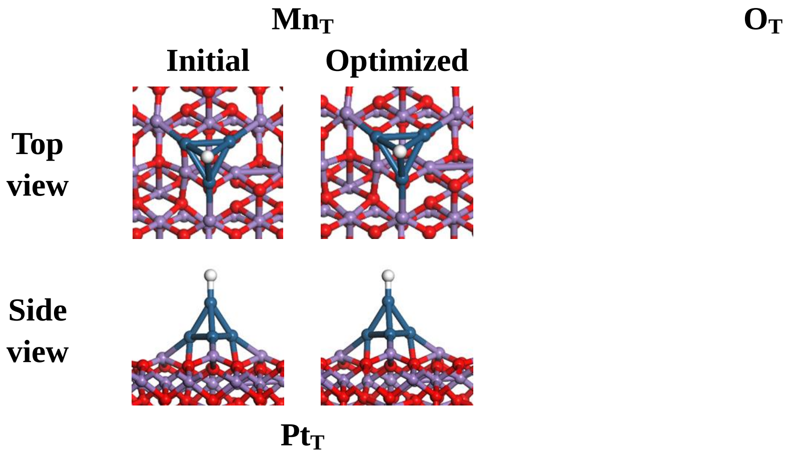

Supplementary Fig S38 | The initial and optimized structures of H adsorbed at different sites on Pt_4/Mn_3O_4 catalyst.

The adsorption behavior of H atom is analyzed on $Pt_4/K_2Mn_4O_8$ catalyst. As listed in Supplementary Table S4, H atom is stably absorbed at the Pt-2_I site with the adsorption energy of -2.00 eV; H atom is stably absorbed at the Mn_H site with the adsorption energy of -1.81 eV; H atom can be stably adsorbed at the O_T site with the adsorption energy of -1.86 eV; H atom is stably adsorbed at the O-1_T, O-2_T and Pt_T sites with the adsorption energy of -1.64, 1.56 and 1.57 eV, respectively; H atom is stably adsorbed at the Mn_T site with the adsorption energy of -0.13 eV; H atom is stably adsorbed at the Pt-Pt_I site with the adsorption energy of -1.63 eV; H atom can be stably adsorbed at the O_I site with the adsorption energy of -1.42 eV. Thus, the most stable site of H atom is the Pt-2_I site; the sub stable site is the O_T site, and the third stable site is the Mn_H site.

The adsorption behavior of H atom is analyzed on Pt_4/Mn_3O_4 catalyst. As listed in Supplementary Table S4, H atom can be stably adsorbed at the Pt-Pt-1_I, Pt-Pt-2_I, Pt-1_I and Pt-2_I sites with the adsorption energies of -5.45, -5.05, -3.99 and -5.26 eV; H atom is stably adsorbed at the Mn_H site with the adsorption energy of -2.58 eV; H atom is stably adsorbed at the Mn-Mn_B site with the adsorption energy of -0.82 eV; H atom is stably adsorbed at the Mn_T site with the adsorption energy of -2.64 eV; H atom is stably adsorbed at the O_T site with the adsorption energy of -3.38 eV; H atom is stably adsorbed at the Pt_T site with the adsorption energy of 0.72 eV. Thus, H atom prefers to be adsorbed at the interface Pt site between Pt_4 cluster and Mn_3O_4 (Pt-Pt-1_I,

Pt-Pt-2_I and Pt-2_I).

Supplementary Table S1 | Pt loadings over different catalysts.

Catalysts	Surface Area (m^2/g)	Pore volume (cm^3/g)	Pore Diameter (nm)
APF	5.96	-	-
Pt NPs/APF-C	6.08	-	-
Pt NPs@MnO_x	28.24	0.08	12.16
Pt NPs/MnO_x	58.36	0.19	13.52
Pt NPs&MnO_x	21.10	0.09	21.26

Supplementary Table S2 | CAL adsorption energy at different adsorption sites on the $Pt_4/K_2Mn_4O_8$, Pt_4/Mn_3O_4 and Pt_4/CeO_2.

	**Adsorption site**	**Adsorption energy (eV)**
**$Pt_4/K_2Mn_4O_8$**	Pt_T with C=C bond	-1.67
	Pt_T with C=O bond	-1.28
	O_V with C=O bond	-2.27
**Pt_4/Mn_3O_4**	Pt_T with C=C bond	-2.19
	Pt-Pt_B with C=C bond	-2.59
	Pt-Mn_I with C=O bond	-2.34
	Pt-Pt_B with C=O bond	-3.47
	Pt-Pt-2_I with C=O bond	-2.32
	O_V with C=O bond	-3.89
**Pt_4/CeO_2**	O_V with C=O bond	-1.17

Supplementary Table S3 | The adsorption energy of COL and HCAL at O_V site on $Pt_4/K_2Mn_4O_8$ catalyst.

	Adsorption species	Adsorption energy (eV)
**$Pt_4/K_2Mn_4O_8$**	COL	-1.21
	HACL	-1.83

Supplementary Table S4 | The adsorption energy of H atom at different adsorption sites over the $Pt_4/K_2Mn_4O_8$ and Pt_4/Mn_3O_4.

	Adsorption site	Adsorption energy (eV)
**$Pt_4/K_2Mn_4O_8$**	Mn_H	-1.81
	$Pt\text{-}2_I$	-2.00
	O_T	-1.86
	$O\text{-}1_T$	-1.64
	$O\text{-}2_T$	-1.56
	Pt_T	-1.57
	$Pt\text{-}Pt_I$	-1.63
	O_I	-1.42
	Mn_T	-0.13
**Pt_4/Mn_3O_4**	$Pt\text{-}Pt\text{-}1_I$	-5.45
	$Pt\text{-}2_I$	-5.26
	$Pt\text{-}Pt\text{-}2_I$	-5.05
	Mn_H	-2.58
	$Pt\text{-}1_I$	-3.99
	$Mn\text{-}Mn_B$	-0.82
	Mn_T	-2.64
	O_T	-3.38
	Pt_T	0.72

Supplementary Table S5 | Selective hydrogenation of CAL over Pt NPs/APF-C, Pt/Mn_xO_y and their mixture.

**Catalysts**[a]	**Conv. (%)**	**Sel. (%)**		
		**COL**	**HCAL**	**HCOL**
Pt NPs/APF-C (10 mg)	24.2	4.4	43.8	51.8
Pt NPs/APF-C+MnO_2 (10 mg+10 mg)	26.2	59.4	23.4	17.2
Pt NPs/APF-C+Mn_2O_3 (10 mg+10 mg)	20.2	67.0	18.0	15.0
Pt NPs/APF-C+Mn_3O_4 (10 mg+10 mg)	27.4	59.7	26.1	14.3
1.4 wt% Pt NPs/MnO_2 (10 mg)	61.3	88.0	6.9	5.1
1.7 wt% Pt NPs/Mn_2O_3 (10 mg)	98.3	80.1	0.6	20.4
1.6 wt% Pt NPs/Mn_3O_4 (10 mg)	88.3	78.0	11.8	10.2

[a]Reaction condition: 70°C, 6 h, 2 MPa H_2

Supplementary Table S6 | Pt loadings over different catalysts.

Catalysts[a]	Pt loadings (wt%)
Pt NPs/APF-C	2.8
Pt NPs@MnO_x-1	4.0
Pt NPs@MnO_x-2	4.4
Pt NPs@MnO_x-3	5.8
Pt NPs@MnO_x	6.1
Pt NPs@MnO_x-5	6.2
Pt NPs/MnO_x	9.4
Pt NPs@MnO_x-Tn	21.3
Pt NPs@MnO_x-Tk	3.3

[a]The results were calculated from ICP-OES data

Reference

1. Wang G H, Hilgert J, Richter F H, *et al.* Platinum-cobalt bimetallic nanoparticles in hollow carbon nanospheres for hydrogenolysis of 5-hydroxymethylfurfural. *Nat Mater* 2014; **13**: 293-300.
2. Kresse G and Furthmüller J *et al.* Efficient iterative schemes for ab initio total-energy calculations using a plane-wave basis set. *Phys Rev B: Condens Matter Mater Phys* 1996; **54**: 11169-86.
3. Delley B *et al.* An all-electron numerical method for solving the local density functional for polyatomic molecules. *J Chem Phys* 1996; **92**: 508-17.
4. Perdew J P, Burke K and Ernzerhof M *et al.* Generalized gradient approximation made simple. *Phys Rev Lett* 1996; **77**: 3865-8.
5. Blochl P E *et al.* Projector augmented-wave method. *Phys Rev B* 1994; **50**: 17953-79.
6. Kresse G and Joubert D *et al.* From ultrasoft pseudopotentials to the projector augmented-wave method. *Phys Rev B* 1999; **59**: 1758-75.
7. Liu F, Rong S, Zhang P, *et al.* One-step synthesis of nanocarbon-decorated MnO_2 with superior activity for indoor formaldehyde removal at room temperature. *Appl Catal B* 2018; **235**: 158-67.
8. Huang Y, Liu Y, Wang W, *et al.* Oxygen vacancy-engineered δ-MnO/activated carbon for room-temperature catalytic oxidation of formaldehyde. *Appl Catal B* 2020; **278**: 119294.
9. Yu D, Ren Y, Yu X, *et al.* Facile synthesis of birnessite-type $K_2Mn_4O_8$ and cryptomelane-type $K_{2-x}Mn_8O_{16}$ catalysts and their excellent catalytic performance for soot combustion with high resistance to H_2O and SO_2. *Appl Catal B* 2021; **285**: 119779.
10. Lu W, Chen J, Kong L, *et al.* Oxygen vacancies modulation Mn_3O_4 nanozyme with enhanced oxidase-mimicking performance for l-cysteine detection. *Sensor Actuat B: Chem* 2021; **333**: 129560.
11. Wu Q, Zhang C, Arai M, *et al.* Pt/TiH_2 catalyst for ionic hydrogenation via stored hydrides in the presence of gaseous H_2. *ACS Catal* 2019; **9**: 6425-34.
12. Wei S, Zhao Y, Fan G, *et al.* Structure-dependent selective hydrogenation of cinnamaldehyde over high-surface-area CeO_2-ZrO_2 composites supported Pt nanoparticles. *Chem Eng J* 2017; **322**: 234-45.
13. Dai Y, Gao X, Chu X, *et al.* On the role of water in selective hydrogenation of cinnamaldehyde to cinnamyl alcohol on PtFe catalysts. *J Catal* 2018; **364**: 192-203.
